# Decarboxylation-Driven Double Annulations: Innovative Multi-Component Reaction Pathways

**DOI:** 10.3390/molecules30071594

**Published:** 2025-04-02

**Authors:** Desheng Zhan, Gang Yang, Tieli Zhou, Sashirekha Nallapati, Xiaofeng Zhang

**Affiliations:** 1 College of Chemistry, Changchun Normal University, Changchun 130032, China; 2 College of Food Science and Engineering, Changchun University, Changchun 130022, China; ygang1221@163.com; 3 Department of Chemistry & Chemical Biology, Northeastern University, 360 Huntington Ave., Boston, MA 02115, USA; 4 Department of Cancer Biology, Dana-Farber Cancer Institute, Harvard Medical School, Harvard University, Boston, MA 02215, USA

**Keywords:** decarboxylation, cycloaddition, annulations, multicomponent reactions, azomethine ylides, converted reactions

## Abstract

A concerted five-component reaction strategy has been developed, featuring double [3+2] cycloadditions utilizing aspartic acid. This approach provides valuable insights into mechanistic pathways, allowing for the distinction between concerted and stepwise processes based on reaction efficiency and diastereoselectivity. Both aspartic and glutamic acids have been employed for a thorough evaluation and exploration of decarboxylation-driven double annulations. This method effectively constructs pyrrolizidine frameworks through a concerted double 1,3-dipolar cycloaddition with aspartic acid, as well as tetrahydropyrrolizinones via three-component double annulations, which include decarboxylative 1,3-dipolar cycloaddition and lactamization with glutamic acid. These highly convergent, decarboxylation-driven multicomponent reactions (MCRs) efficiently produce fused polyheterocyclic systems while being environmentally friendly, generating only CO_2_ and water as byproducts.

## 1. Introduction

The recent advancements in green synthetic chemistry have emphasized the development of multicomponent reactions (MCRs) due to their remarkable efficiency in constructing complex molecular architectures from simple starting materials [1,2,3,4,5,6]. Among these, reactions that incorporate cycloadditions/cyclizations have garnered significant attention for their ability to build polycyclic structures, crucial in the design of biologically active compounds [7,8,9,10,11,12,13]. Specifically, a novel decarboxylation-driven five-component reaction (5-CR) involving a concerted double [3+2] cycloadditions with the tolerance of various α-amino acids [14,15,16] difference with one-pot two-step double [3+2] cycloadditions with α-amino esters [17,18,19,20] offers a powerful tool for constructing intricate pyrrolizidine frameworks, which are renowned for their presence in a diversity of natural alkaloids with notable biological activities. Pyrrolizidine alkaloids are widely recognized for their diverse pharmacological properties, including antitumor, antiviral, and anti-inflammatory activities [21,22,23,24]. As such, the synthesis of pyrrolizidine and related structures continues to be of substantial interest in the development of therapeutic agents [25,26,27,28]. Our novel methodology leverages the reactivity of α-amino acids to initiate double 1,3-dipolar cycloadditions, providing a versatile platform to explore these biologically relevant scaffolds.

In our ongoing research on decarboxylation-driven five-component reactions (5-CR) of α-amino acids through double [3+2] cycloadditions [14,15,16], the strategic synthesis of pyrrolizidines using aspartic acid underscores the efficiency and diastereoselectivity of this concerted double 1,3-dipolar cycloadditions process. Compared to the stepwise method, this approach reveals distinct differences in reaction mechanisms between concerted and stepwise double [3+2] cycloadditions. This concerted 5-CR not only enhances diastereoselective outcomes but also streamlines the overall reaction pathway for constructing pyrrolizidines, surpassing the stepwise approach by a significant margin. In addition, this one-step double-annulations strategy enables the efficient synthesis of tetrahydropyrrolizinones through a decarboxylative three-component reaction (3-CR) utilizing glutamic acid. It is achieved via a sequential mechanism involving 1,3-dipolar cycloaddition followed by lactamization in line with the rapid synthesis of tetrahydropyrrolothiazoles with cysteine via a decarboxylative four-component reaction (4-CR) [29]. These two MCR examples, embodying the pot, atom, step, and economic (PASE) approach [30,31], not only optimize the yield and diastereoselectivity but also highlight the environmentally friendly nature of the process, generating only CO_2_ and water as byproducts [32,33].

In this article, we explore the mechanistic insights contrasting concerted and stepwise processes, providing a detailed account of the comprehensive evaluation undertaken to harness the potential of aspartic and glutamic acids in decarboxylation-driven double annulations. By elucidating the nuanced mechanistic pathways distinguishing concerted from stepwise processes, we aim to lay the groundwork for the rapid advancement and application of MCRs in green and medicinal chemistry and beyond.

## 2. Results and Discussion

The initial optimization of the reaction was conducted using benzaldehyde **1a**, N-ethyl maleimide **2a**, and aspartic acid **3a** as substrates, with AcOH serving as the protonic source. After evaluating a series of solvents, reaction times, and temperatures, it was observed that using 1.0 equivalent of AcOH significantly increased the LC yield of compound **4a** to a range of 39–71% (Table 1). The optimal conditions for the one-step synthesis of compound **4a** were determined to be a molar ratio of 2.1:2.0:1.1 for **1a**:**2a**:**3a** in ethanol (EtOH) at 110 °C for 12 h, resulting in a 71% LC yield and a 66% isolated yield (entry 3).

**Figure 1:**
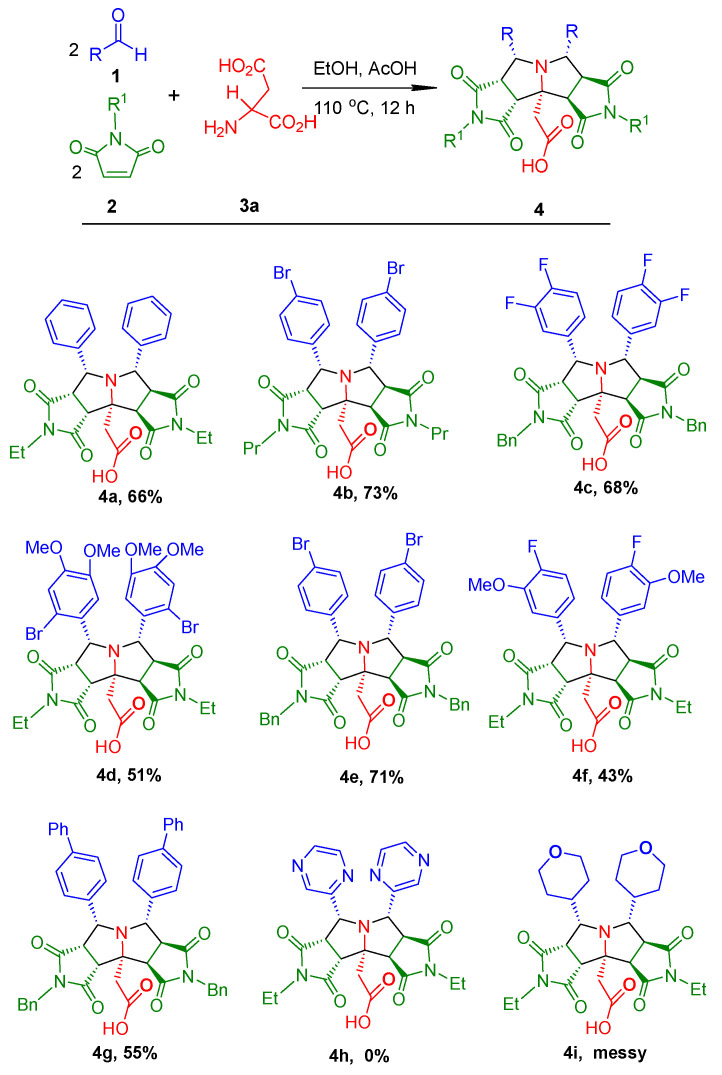
One-step synthesis of pyrrolizidines **4**.

Under the optimized reaction conditions, a five-component reaction was conducted using various aldehydes **1**, maleimides **2**, and aspartic acid **3a** to synthesize pyrrolizidines **4** with a range of substituents (Figure 1). Compounds **4a**–**4g**, incorporating both electron-donating and electron-withdrawing groups, were obtained in isolated yields ranging from 43% to 73%. Notably, pyrazine aldehydes did not produce the expected product **4h**, likely due to the increased basicity of the reaction environment. Additionally, when aromatic aldehydes were replaced with aliphatic aldehydes like tetrahydro-2H-pyran-4-carbaldehyde, the reaction yielded complex mixtures of diastereomers due to the alkyl groups, without localizing the negative charge on the carbon in the 1,3-dipoles, compared to the electron-withdrawing (EWG) or aromatic groups, exemplified by compound **4i**. This outcome suggests that aliphatic aldehydes introduce considerable stereochemical challenges in decarboxylative double [3+2] cycloadditions [14,29,32]. The stereochemistry of pyrrolizidines **4** was identified by X-ray crystallography in our previous work [14].

**Scheme 1:**
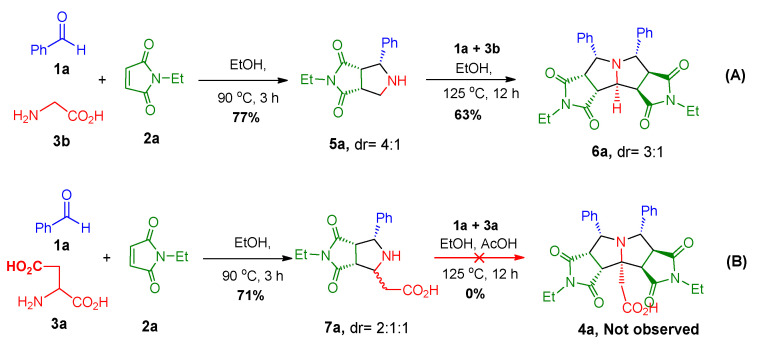
Comparison of glycine and aspartic acid in stepwise double [3+2] cycloadditions.

In our previous work, we developed a novel five-component reaction (5-CR) via double [3+2] cycloadditions to synthesize multi-substituted pyrrolizidines using eight α-amino acids [14]. This approach allowed us to systematically investigate and elucidate the reaction mechanism through a stepwise process. To refine the reaction conditions for the stepwise method, we used glycine as a model substrate (Scheme 1A). In the first step, a decarboxylative [3+2] cycloaddition afforded pyrrolidine **5a** with an isolated yield of 77% and a diastereomeric ratio (dr) of **4:1**. Subsequently, **5a** underwent a second [3+2] cycloaddition via α-C-H functionalization of the new N-H pyrrolidine ring [34,35], yielding product **6a** with 63% isolated yield and dr of 3:1. However, the reaction efficiency and stereoselectivity in this stepwise approach were significantly lower than those observed in one-step and five-component double [3+2] cycloadditions, highlighting the advantages of the latter in terms of yield and selectivity [14].

In this study, we aimed to rigorously validate the reaction mechanism of decarboxylative double [3+2] cycloadditions through systematic experimental investigations. This comprehensive research offers critical insights into the mechanistic intricacies of 5-CR, thereby establishing a robust foundation for future research endeavors. Utilizing aspartic acid as a model substrate, we attempted the stepwise synthesis of compound **4a** (Scheme 1B). However, the decarboxylative [3+2] cycloaddition predominantly resulted in a mixture of diastereomers **7a**. Subsequent efforts to obtain compound **4a** via various experimental pathways were unsuccessful. This outcome is consistent with our previous findings in the synthesis of bispiro[oxindole-pyrrolidine]s [15,16].

**Scheme 2:**
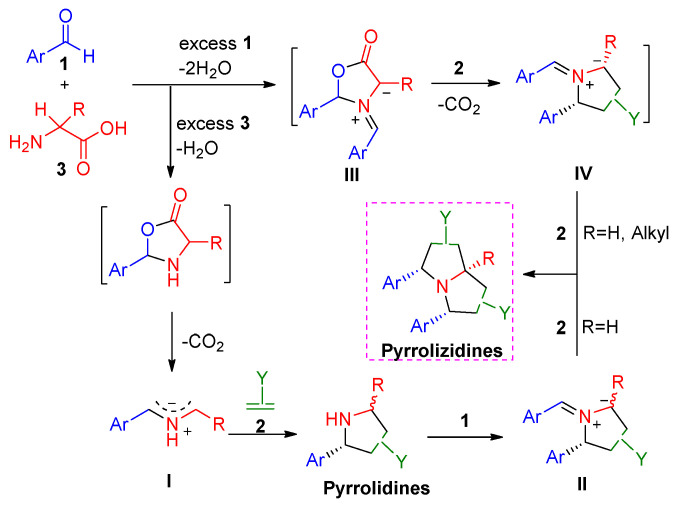
Two different mechanistic pathways of double [3+2] cycloadditions.

Our findings indicated that the stepwise approach often fails to yield the desired pyrrolizidines with α-amino acids except for glycine [14,15,16], while it is efficiently achieved through one-step double [3+2] cycloadditions. Additionally, even in instances where the one-pot or stepwise approach with glycine succeeds, its stereoselectivity is significantly lower compared to the 5-CR process. These outcomes provide compelling evidence that the reaction mechanism of this novel five-component transformation is most likely a concerted process rather than a tandem reaction. The plausible mechanism proposed (Scheme 2): the stepwise reaction accomplished by the usage of excess amino acids offers a semi-stabilized azomethine ylide (AMY) **(I)** to form the new pyrrolidines with less stereoselectivity under purification; then, the second semi-stabilized AMY **(II)** are generated by the α-C-H functionalization of new pyrrolidines N-H to support the synthesis of bicyclic pyrrolizidines, which is only feasible while R = H, otherwise high steric hindrance of R would block the occurrence of α-C-H functionalization. The one-step 5-CR through a concerted double [3+2] cycloadditions is achieved by utilizing excess amounts of compounds **1** and **3** (two equivalents) relative to amino acids, which leads to the formation of stabilized AMY **(III)** incorporating an electron-withdrawing lactone group to facilitate the construction of diastereoselective pyrrolidines along with the formation of the semi-stabilized AMY **(IV)** via decarboxylation, which is used to synthesize highly diastereoselective pyrrolizidines (Scheme 2). This method offers the advantage of substrate diversity, accommodating both hydrogen and alkyl substituents (R = H, alkyl). Thus, the mechanistic distinction between the stepwise and concerted double [3+2] cycloadditions provides a compelling explanation for the former’s low efficiency and stereoselectivity due to two sequential semi-stabilized AMYs **(I)** and **(II)**, with its applicability restricted mainly to glycine. Consequently, for the past 40 years, decarboxylative [3+2] cycloadditions have predominantly originated from N-dialkylglycines, largely because the concerted mechanism has remained elusive [32,36,37,38,39,40].

**Figure 2:**
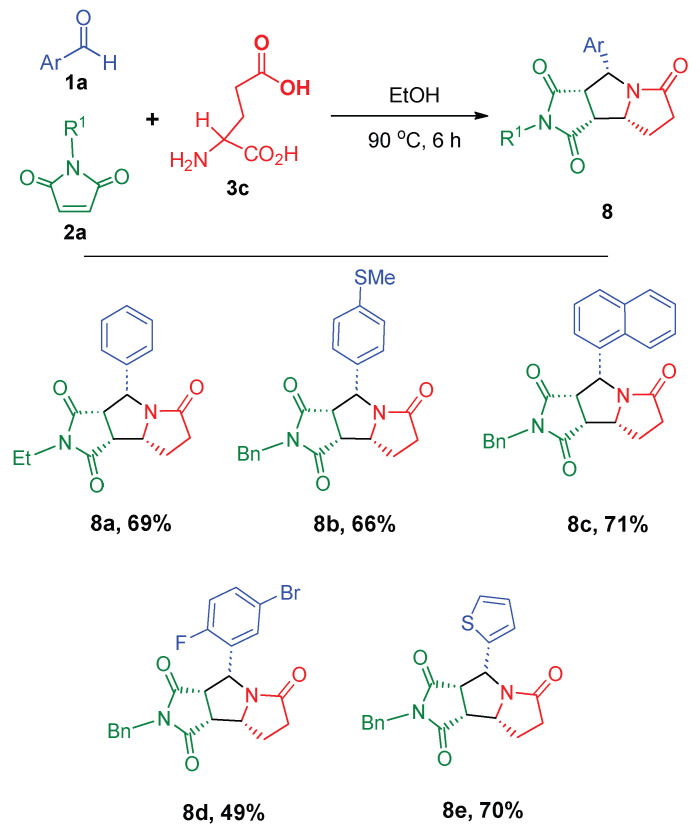
One-step synthesis of tetrahydropyrrolizinones **8**.

**Scheme 3:**
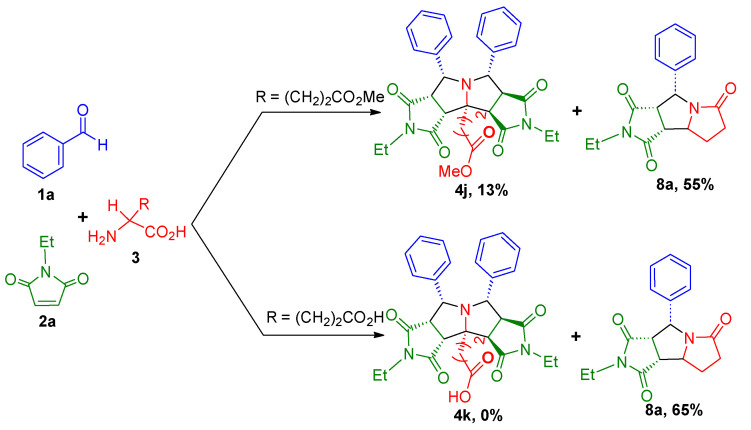
The three-component double annulations with glutamic acid and ester.

**Scheme 4:**
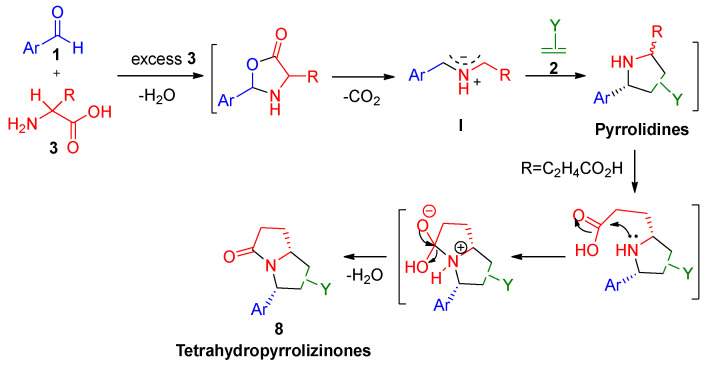
The proposed mechanism of double annulations with glutamic acid.

To further evaluate the diverse function of α-substituted amino acids in decarboxylative [3+2] cycloaddition-initiated double annulations, we performed glutamic acid and ester (**3c** and **3d**) in the replacement of aspartic acid. These results demonstrated that the reaction conditions (Table 1, entry 3) only gave a 13% isolated yield of compound **4j**, but failed to afford product **4k** (Scheme 3). Surprisingly, compound **8a** with a 55% and 65% isolated yield was obtained through a three-component reaction (3-CR) incorporating decarboxylative 1,3-dipolar cycloaddition followed by lactamization. Under the optimal reaction conditions, 1.1:1.0:1.5 of **1a**:**2a**:**3c** in EtOH (0.2 M) at 90 °C for 6 h, we further explored a novel 3-CR in the readily synthesis of tetrahydropyrrolizinones **8a–e** with 49 to 71% isolated yield and ~dr 3:1 (Figure 2). The plausible mechanism of this 3-CR shows in Scheme 4, a semi-stabilized AMY **(I)** generated through the decarboxylation of excess **3c** reacting with aldehydes **1**, that a [3+2] cycloaddition with dipolarophiles **2** to form pyrrolidines, then followed by lactamization for the synthesis of tetrahydropyrrolizinones (Scheme 4). Interestingly, we failed to capture pyrrolidines from decarboxylative 1,3-dipolar cycloaddition with glutamic acid, even in mild reaction conditions. Therefore, we currently lack evidence to confirm whether this double annulation is a concerted or tandem reaction process. This represents an intriguing area for future chemical research.

## 3. Materials and Methods

Chemicals and solvents were purchased from Sigma, TCI, and Oakwood and were of the highest purity available and used without further purification. ^1^H NMR (400 MHz) and ^13^C NMR spectra (101 MHz) were recorded on Bruker NMR spectrometers. Chemical shifts were reported in parts per million (ppm). LC–MS was performed on an Agilent 2100 LC with 6130 quadrupole MS spectrometers. A C18 column (5.0 μm, 6.0 × 50 mm) was used for the separation. The mobile phases were MeCN and H_2_O, both containing 0.01% HCO_2_H. Low-resolution mass spectra were recorded in APCI (atmospheric pressure chemical ionization). Flash chromatography separations were performed on Biotage flash column system with silica gel columns (230–400 μm mesh).

General procedure of five-component reaction for compounds **4**: To a solution of aldehyde **1** (2.1 mmol) and aspartic acid **3a** (1.1 mmol) with 5.0 mL of EtOH in the sealed tube was added maleimide **2** (2.0 mmol) and AcOH (1.0 mmol) after being stirred at 110 °C for 12 h. Upon the completion of the reaction as monitored by LC–MS, the concentrated reaction mixture was isolated on a semi-preparative HPLC with a C18 column. Product **4** was afforded.

General procedure of the three-component reaction for compounds **8**: To a solution of aldehyde **1** (1.1 mmol) and glutamic acid **3c** (1.5 mmol) with 10.0 mL of EtOH in the sealed tube was added maleimide **2** (1.0 mmol) after being stirred at 90 °C for 6 h. Upon the completion of the reaction as monitored by LC–MS, the mixture residue was isolated on a semi prep-HPLC with C18 column to afford product **8**.

Compound **4a**, off-white solid, 66% isolated yield. ^1^H NMR (400 MHz, CDCl_3_) δ 7.04 (d, J = 6.9 Hz, 2H), 6.97–6.86 (m, 5H), 6.72 (dd, J = 6.4, 2.8 Hz, 2H), 4.84 (d, J = 9.0 Hz, 1H), 4.65 (d, J = 8.4 Hz, 1H), 4.49 (d, J = 10.8 Hz, 1H), 3.82 (d, J = 8.1 Hz, 1H), 3.70 (ddd, J = 17.5, 11.2, 5.6 Hz, 3H), 3.50–3.36 (m, 2H), 3.24 (ddd, J = 40.1, 23.6, 11.8 Hz, 3H), 3.02 (d, J = 17.0 Hz, 1H), 1.36 (t, J = 7.2 Hz, 3H), 0.90 (t, J = 7.2 Hz, 3H). ^13^C NMR (101 MHz, CDCl_3_) δ 176.4, 176.3, 176.1, 173.5, 137.7, 131.3, 130.5, 128.5, 127.5, 127.5, 127.2, 126.8, 74.5, 68.6, 65.8, 54.2, 49.5, 49.0, 47.9, 40.97, 34.2, 33.9, 12.6, 12.3. HRMS (EI, *m*/*z*): calcd. for C_29_H_29_N_3_O_6_: 515.2056, Found: 515.2060.

Compound **4b**, off-white solid, 73% isolated yield. ^1^H NMR (400 MHz, DMSO-*d*_6_) δ 12.41 (s, 1H), 7.03 (dd, J = 14.7, 8.5 Hz, 4H), 6.93 (d, J = 8.6 Hz, 2H), 6.67 (d, J = 8.5 Hz, 2H), 4.85 (d, J = 9.1 Hz, 1H), 4.49 (d, J = 8.4 Hz, 1H), 4.29 (d, J = 10.7 Hz, 1H), 3.88 (d, J = 8.2 Hz, 1H), 3.70–3.63 (m, 1H), 3.42 (td, J = 13.5, 6.0 Hz, 2H), 3.28–3.12 (m, 3H), 3.03–2.92 (m, 2H), 1.62 (dd, J = 14.9, 7.5 Hz, 2H), 1.12 (dt, J = 14.4, 7.3 Hz, 2H), 0.94 (t, J = 7.4 Hz, 3H), 0.66 (t, J = 7.4 Hz, 3H). ^13^C NMR (101 MHz, DMSO-*d*_6_) δ 177.9, 177.7, 176.2, 174.3, 172.9, 139.3, 134.1, 132.2, 130.3, 130.0, 129.6, 121.5, 119.6, 74.5, 66.5, 64.4, 54.4, 49.6, 48.8, 47.8, 20.8, 20.4, 11.9, 11.7. HRMS (EI, *m*/*z*): calcd. for C_31_H_31_Br_2_N_3_O_6_: 699.0580, Found: 699.0576.

Compound **4c**, white solid, 68% isolated yield. ^1^H NMR (400 MHz, DMSO-*d*_6_) δ 12.38 (s, 1H), 7.56–7.43 (m, 4H), 7.26–7.14 (m, 3H), 7.05–6.91 (m, 2H), 6.91–6.78 (m, 1H), 6.73–6.53 (m, 3H), 6.09 (s, 1H), 5.93 (s, 1H), 4.83 (d, J = 9.4 Hz, 1H), 4.71–4.51 (m, 3H), 4.20 (q, J = 14.8 Hz, 2H), 3.97 (d, J = 8.1 Hz, 1H), 3.76–3.67 (m, 1H), 3.59 (d, J = 10.5 Hz, 1H), 3.18–3.06 (m, 2H), 2.40 (d, J = 17.7 Hz, 1H). ^13^C NMR (101 MHz, DMSO-*d*_6_) δ 177.2, 177.0, 175.9, 173.9, 172.7, 149.4, 146.8, 145.4, 136.9, 136.1, 135.7, 130.2, 129.6, 129.5, 128.8, 128.6, 127.9, 127.7, 121.2, 121.1, 116.3, 116.1, 116.0, 115.8, 74.4, 65.9, 63.7, 54.2, 49.4, 48.9, 47.6, 42.5, 41.9, 41.1. HRMS (EI, *m*/*z*): calcd. for C_39_H_29_F_4_N_3_O_6_: 711.1992, Found: 711.1988.

Compound **4d**, off-white solid, 51% isolated yield. ^1^H NMR (400 MHz, DMSO-*d*_6_) δ 12.28 (s, 1H), 6.89 (s, 1H), 6.76 (s, 1H), 6.68 (s, 1H), 6.54 (s, 1H), 5.12 (dd, J = 5.9, 2.2 Hz, 1H), 4.47 (d, J = 8.4 Hz, 1H), 4.26 (d, J = 10.6 Hz, 1H), 4.07 (d, J = 5.2 Hz, 1H), 3.86–3.79 (m, 2H), 3.67 (s, 3H), 3.56 (s, 3H), 3.51 (s, 3H), 3.34–3.20 (m, 3H), 3.13 (d, J = 5.0 Hz, 3H), 3.06 (d, J = 7.2 Hz, 2H), 2.58 (d, J = 17.7 Hz, 1H), 1.20 (t, J = 7.2 Hz, 3H), 0.74 (t, J = 7.2 Hz, 3H). ^13^C NMR (101 MHz, DMSO-*d*_6_) δ 178.4, 177.6, 176.0, 173.6, 172.7, 149.1, 148.1, 147.4, 147.0, 130.4, 124.3, 117.8, 116.0, 115.0, 114.6, 113.1, 111.0, 73.7, 65.5, 64.0, 56.4, 56.2, 55.7, 55.4, 54.4, 49.0, 48.0, 47.6, 47.4, 34.2, 33.2, 13.4, 13.1. APCIMS *m*/*z*: 794.2 (M^+^ + 1).

Compound **4e**, off-white solid, 71% isolated yield. ^1^H NMR (400 MHz, DMSO-*d*_6_) δ 12.33 (s, 1H), 7.59–7.44 (m, 5H), 7.25–7.16 (m, 3H), 6.95 (dd, J = 6.6, 3.0 Hz, 2H), 6.85 (d, J = 8.6 Hz, 2H), 6.71 (d, J = 8.5 Hz, 2H), 6.53 (d, J = 8.6 Hz, 2H), 6.01 (d, J = 8.5 Hz, 2H), 4.77 (d, J = 9.4 Hz, 1H), 4.69–4.51 (m, 3H), 4.18 (q, J = 14.8 Hz, 2H), 3.95 (d, J = 8.1 Hz, 1H), 3.72–3.59 (m, 2H), 3.08 (dd, J = 10.6, 8.6 Hz, 2H), 2.38 (d, J = 17.7 Hz, 1H). ^13^C NMR (101 MHz, DMSO-*d*_6_) δ 177.2, 176.9, 176.0, 173.9, 172.8, 138.4, 136.1, 135.6, 134.2, 131.8, 130.0, 129.7, 129.5, 129.2, 128.9, 128.6, 128.0, 127.7, 121.5, 119.2, 74.4, 66.7, 64.2, 54.2, 49.5, 49.2, 49.0, 47.6, 42.4, 41.8. HRMS (EI, *m*/*z*): calcd. for C_39_H_31_Br_2_N_3_O_6_: 795.0580, Found: 795.0577.

Compound **4f**, off-white solid, 43% isolated yield. ^1^H NMR (400 MHz, DMSO-*d*_6_) δ 12.25 (s, 1H), 6.76–6.70 (m, 2H), 6.69–6.62 (m, 2H), 6.53 (dd, J = 8.6, 1.9 Hz, 1H), 6.36 (ddd, J = 8.3, 4.4, 1.9 Hz, 1H), 4.85 (d, J = 9.1 Hz, 1H), 4.46 (d, J = 8.4 Hz, 1H), 4.30 (d, J = 10.7 Hz, 1H), 3.83 (d, J = 8.1 Hz, 1H), 3.73–3.64 (m, 1H), 3.61 (s, 3H), 3.59–3.47 (m, 5H), 3.27–3.17 (m, 2H), 3.06 (q, J = 7.1 Hz, 2H), 2.58 (d, J = 17.7 Hz, 1H), 1.18 (dd, J = 12.8, 5.6 Hz, 3H), 0.74 (t, J = 7.2 Hz, 3H). ^13^C NMR (101 MHz, DMSO-*d*_6_) δ 177.7, 177.5, 176.0, 174.1, 172.9, 152.3, 151.3, 149.8, 148.9, 145.8, 145.7, 145.5, 145.4, 136.8, 134.9, 129.8, 129.8, 124.9, 124.8, 120.0, 117.7, 114.8, 114.6, 114.4, 113.0, 74.0, 66.7, 64.3, 55.9, 55.8, 54.7, 49.5, 49.0, 48.9, 47.9, 33.9, 33.1, 13.0, 12.4. APCIMS *m*/*z*: 612.2 (M^+^ + 1).

Compound **4g**, white solid, 55% isolated yield. ^1^H NMR (400 MHz, DMSO-*d*_6_) δ 12.33 (s, 1H), 7.61–7.49 (m, 5H), 7.31–7.13 (m, 12H), 7.00 (dd, J = 7.1, 2.1 Hz, 2H), 6.83 (d, J = 8.3 Hz, 2H), 6.72 (dd, J = 19.8, 8.4 Hz, 4H), 6.16 (d, J = 8.4 Hz, 2H), 4.85 (d, J = 9.4 Hz, 1H), 4.68 (dd, J = 23.3, 13.8 Hz, 2H), 4.59 (d, J = 8.5 Hz, 1H), 4.22 (d, J = 1.6 Hz, 2H), 3.99 (d, J = 8.1 Hz, 1H), 3.75 (dd, J = 17.0, 9.4 Hz, 2H), 3.23 (d, J = 17.9 Hz, 1H), 3.14 (dd, J = 10.5, 8.6 Hz, 1H), 2.51–2.45 (m, 2H). ^13^C NMR (101 MHz, DMSO-*d*_6_) δ 177.4, 177.2, 176.2, 174.1, 172.9, 140.3, 140.2, 139.8, 138.4, 137.8, 136.2, 135.6, 132.6, 131.6, 129.8, 129.5, 128.9, 128.6, 128.1, 127.7, 127.5, 127.2, 127.0, 126.7, 125.4, 125.0, 74.3, 67.4, 64.8, 54.4, 49.7, 49.2, 47.8. APCIMS *m*/*z*: 792.3 (M^+^ + 1).

Compound **8a**, white solid, 69% isolated yield. ^1^H NMR (400 MHz, CDCl_3_) δ 7.29 (dd, J = 12.3, 4.6 Hz, 1H), 7.25–7.18 (m, 2H), 7.11 (d, J = 6.7 Hz, 2H), 5.61 (d, J = 9.6 Hz, 1H), 4.78–4.69 (m, 1H), 3.90 (t, J = 9.5 Hz, 1H), 3.24 (dd, J = 9.4, 4.4 Hz, 1H), 3.15 (dt, J = 13.3, 6.7 Hz, 2H), 2.81–2.66 (m, 2H), 2.48 (ddd, J = 17.0, 8.9, 4.8 Hz, 1H), 2.03–1.89 (m, 1H), 0.53 (t, J = 7.2 Hz, 3H). ^13^C NMR (101 MHz, CDCl_3_) δ 176.1, 174.8, 173.2, 138.9, 136.2, 129.0, 128.5, 128.2, 127.9, 126.9, 125.5, 62.9, 61.5, 59.3, 58.9, 55.3, 52.8, 52.4, 47.1, 34.3, 33.6, 33.5, 32.6, 29.9, 20.6, 12.8, 12.0. HRMS (EI, *m*/*z*): calcd. for C_17_H_18_N_2_O_3_: 298.1317, Found: 298.1321.

Compound **8b**, white solid, 66% isolated yield. ^1^H NMR (400 MHz, CDCl_3_) δ 7.24–7.13 (m, 3H), 7.02–6.86 (m, 6H), 5.51 (d, J = 9.9 Hz, 1H), 4.57 (dd, J = 12.9, 6.8 Hz, 1H), 4.30 (dd, J = 43.1, 14.0 Hz, 2H), 3.96 (t, J = 9.8 Hz, 1H), 3.25 (dd, J = 9.7, 5.2 Hz, 1H), 2.78–2.63 (m, 2H), 2.50–2.42 (m, 1H), 2.41 (d, J = 0.7 Hz, 3H), 1.96 (dt, J = 10.7, 6.1 Hz, 1H). ^13^C NMR (101 MHz, CDCl_3_) δ 175.8, 174.4, 173.0, 138.6, 134.8, 132.6, 128.7, 128.5, 127.9, 127.1, 126.1, 63.0, 58.3, 52.9, 52.4, 42.5, 33.4, 29.3, 15.4. HRMS (EI, *m*/*z*): calcd. for C_23_H_22_N_2_O_3_S: 406.1351, Found: 406.1357.

Compound **8c**, white solid, 71% isolated yield. ^1^H NMR (400 MHz, CDCl_3_) δ 8.15 (d, J = 8.5 Hz, 1H), 7.81 (dd, J = 8.1, 0.7 Hz, 1H), 7.67 (d, J = 8.2 Hz, 1H), 7.60 (ddd, J = 8.4, 6.9, 1.3 Hz, 1H), 7.56–7.47 (m, 1H), 7.24–7.03 (m, 4H), 6.95 (d, J = 7.1 Hz, 1H), 6.87–6.80 (m, 2H), 6.34 (d, J = 9.9 Hz, 1H), 4.68 (dd, J = 13.2, 7.0 Hz, 1H), 4.29–4.05 (m, 3H), 3.31 (dd, J = 9.5, 5.6 Hz, 1H), 2.87–2.69 (m, 2H), 2.53 (ddd, J = 12.4, 7.0, 4.5 Hz, 1H), 2.06 (ddd, J = 16.0, 12.9, 12.0 Hz, 1H). ^13^C NMR (101 MHz, CDCl_3_) δ 175.4, 174.7, 172.3, 134.9, 133.4, 133.0, 131.2, 129.0, 128.61, 128.4, 127.8, 126.1, 124.8, 124.1, 122.1, 63.8, 55.7, 52.9, 52.2, 42.2, 33.5, 29.2. HRMS (EI, *m*/*z*): calcd. for C_26_H_22_N_2_O_3_: 410.1630, Found: 410.1632.

Compound **8d**, white solid, 49% isolated yield. ^1^H NMR (400 MHz, CDCl_3_) δ 7.32–7.27 (m, 1H), 7.27–7.23 (m, 3H), 7.17–7.09 (m, 3H), 6.76 (t, J = 9.3 Hz, 1H), 5.56 (d, J = 10.3 Hz, 1H), 4.52–4.37 (m, 3H), 4.04 (t, J = 10.0 Hz, 1H), 3.23 (dd, J = 9.6, 6.5 Hz, 1H), 2.79–2.61 (m, 2H), 2.52–2.42 (m, 1H), 2.11–1.97 (m, 1H). ^13^C NMR (101 MHz, CDCl_3_) δ 174.9, 174.8, 172.8, 160.7, 158.2, 134.9, 133.0, 132.9, 130.6, 128.8, 128.7, 128.1, 126.4, 126.2, 117.4, 117.1, 116.8, 116.8, 63.5, 63.5, 54.2, 52.6, 51.6, 42.6, 33.3, 28.5. HRMS (EI, *m*/*z*): calcd. for C_22_H_18_BrFN_2_O_3_: 456.0485, Found: 456.0479,

Compound **8e**, white solid, 70% isolated yield. ^1^H NMR (400 MHz, CDCl_3_) δ 7.25 (d, J = 1.2 Hz, 1H), 7.24–7.20 (m, 2H), 7.14–7.08 (m, 2H), 7.00–6.95 (m, 1H), 6.87–6.82 (m, 1H), 6.77 (ddd, J = 4.9, 3.6, 1.1 Hz, 1H), 5.83 (d, J = 9.4 Hz, 1H), 4.60 (dd, J = 12.7, 6.8 Hz, 1H), 4.35 (dd, J = 37.1, 14.0 Hz, 2H), 3.92 (t, J = 9.6 Hz, 1H), 3.23 (dd, J = 9.8, 5.4 Hz, 1H), 2.76–2.64 (m, 2H), 2.50–2.39 (m, 1H), 2.02–1.94 (m, 1H). ^13^C NMR (101 MHz, CDCl_3_) δ 175.6, 173.9, 172.6, 138.5, 134.8, 128.9, 128.5, 127.9, 127.1, 126.7, 124.8, 61.8, 54.4, 52.8, 52.8, 42.5, 33.1, 28.6. APCIMS *m*/*z*: 367.2 (M^+^ + 1).

## 4. Conclusions

In conclusion, our study introduces a 5-CR featuring double [3+2] cycloadditions utilizing aspartic acid to construct pyrrolizidines along with investigation and highlights of the superior efficiency and diastereoselectivity through concerted reaction process. Additionally, we demonstrate a 3-CR with glutamic acid that incorporates decarboxylative 1,3-dipolar cycloaddition followed by lactamization to synthesize tetrahydropyrrolizinones. By elucidating the mechanistic pathways of decarboxylation-driven double [3+2] cycloadditions, distinguishing concerted from stepwise processes, we provide valuable insights into a novel 5-CR reaction mechanism and highlight two types of multicomponent reactions (MCRs). This paves the way for efficient construction of fused polyheterocyclic systems. Our methodology optimizes both yield and synthetic utility while adhering to the principles of pot, atom, step, and economic (PASE) chemistry. The use of aspartic and glutamic acids, which results in only CO_2_ and water as byproducts, underscores the environmentally friendly nature of the process. We believe that the double-annulations strategy will inspire further exploration into the development of sustainable synthetic methodologies and broaden the scope of applications in medicinal chemistry and beyond.

## Figures and Tables

**Table 1:**
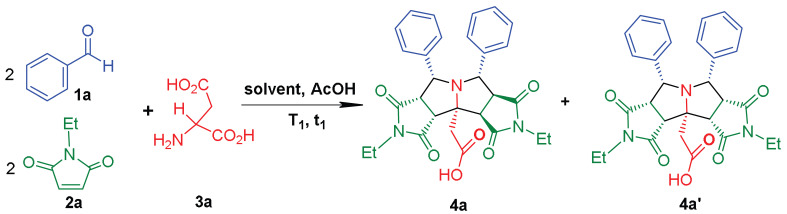
Optimization of reaction conditions with aspartic acid **3a** ^a^.

Entry	Solvent	T_1_ (°C)	t_1_ (h)	4a (%) ^b^	Dr ^c^
1	EtOH	90	6	53	4:1
2	EtOH	90	12	60	4:1
3	EtOH	110	12	71 (66)	4:1
4	EtOH	125	12	63	4:1
5 ^d^	EtOH	110	12	55	3:1
6	MeCN	110	12	46	4:1
7	MeOH	85	24	39	3:1

^a^ **1a**:**2a**:**3a** = 2.1:2.0:1.1; ^b^ LC yield, isolated yield in parenthesis; ^c^ 4a:4a’ dr before separation; ^d^ AcOH not added.

## Data Availability

Data is contained within the article or Supplementary Materials.

## Supplementary Materials

The following supporting information can be downloaded at: https://www.mdpi.com/article/10.3390/molecules30071594/s1, including ^1^H-NMR and ^13^C-NMR of final products.
