# Supplementary material for: Decarboxylation-Driven Double Annulations: Innovative Multi-Component Reaction Pathways
Source: Molecules. 2025 Apr 2;30(7):1594. doi: 10.3390/molecules30071594 (PMC11990421; doi:10.3390/molecules30071594)
Supplement: Supplementary file 1 [file molecules-30-01594-s001.zip › molecules-3550088-supplementary.pdf]

Supplementary Materials

Decarboxylation-Driven Double Annulations: Innovative Multi-Component Reaction Pathways

Desheng Zhan [1], Gang Yang [2], Tieli Zhou [2,*], Sashirekha Nallapati [3] and Xiaofeng Zhang [4,*]

[1] College of Chemistry, Changchun Normal University, Changchun 130032, China
[2] College of Food Science and Engineering, Changchun University, Changchun 130022, China
[3] Department of Chemistry & Chemical Biology, Northeastern University, 360 Huntington Ave, Boston, MA 02115, USA
[4] Department of Cancer Biology, Dana-Farber Cancer Institute, Harvard Medical School, Harvard University, Boston, MA 02215, USA

S1. Experimental

Chemicals and solvents were purchased from Sigma, TCI, and Oakwood and were of the highest purity available and used without further purification. ^{1}H NMR (400 MHz) and ^{13}C NMR spectra (101 MHz) were recorded on Bruker NMR spectrometers. Chemical shifts were reported in parts per million (ppm). LC–MS was performed on an Agilent 2100 LC with 6130 quadrupole MS spectrometers. A C18 column (5.0 µm, 6.0 x 50 mm) was used for the separation. The mobile phases were MeCN and H_2O, both containing 0.01% HCO_2H. Low-resolution mass spectra were recorded in APCI (atmospheric pressure chemical ionization). Flash chromatography separations were performed on Biotage flash column system with silica gel columns (230-400 µm mesh).

S2. General procedures

S2.1. General procedure for the one-step synthesis of compounds 4

To a solution of aldehyde **1** (2.1 mmol) and aspartic acid **3a** (1.1 mmol), in 5.0 mL of EtOH was added maleimide **2** (2.0 mmol) after being stirred at 110 °C for 12 h. Upon the completion of the reaction as monitored by LC–MS, the concentrated reaction mixture was isolated on a semi-preparative HPLC with a C18 column. Product **4** was afforded.

S2.2. General procedure for the one-step synthesis of compounds 8

To a solution of aldehyde **1** (1.1 mmol) and glutamic acid **3c** (1.5 mmol), in 10.0 mL of EtOH was added maleimide **2** (1.0 mmol) after being stirred at 90 °C for 6 h. Upon the completion of the reaction as monitored by LC–MS, the mixture residue was isolated on a semi prep-HPLC with C18 column to afford product **8**.

S3. NMR Spectra of Products 4 and 8

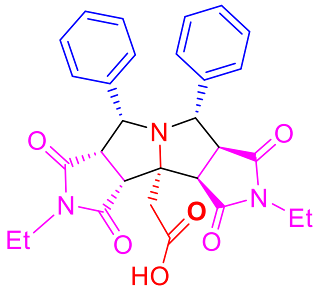
O
N
O
N
Et
O
HO
O
O
N
Et

4a

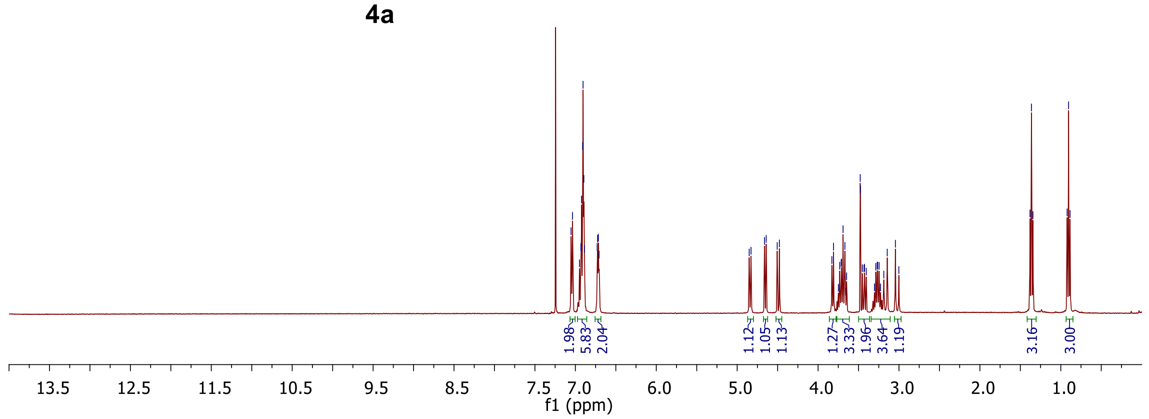
7.05
7.04
6.95
6.93
6.92
6.91
6.90
6.90
6.89
6.73
6.72
6.72
6.71
4.85
4.83
4.66
4.64
4.51
4.48
3.83
3.81
3.71
3.71
3.69
3.67
3.48
3.48
3.27
3.14
3.04
1.38
1.36
1.34
0.92
0.90
0.89
1.98
5.83
2.04
1.12
1.05
1.13
1.27
3.33
1.96
3.64
1.19
3.16
3.00
13.5
12.5
11.5
10.5
9.5
8.5
7.5
7.0
6.0
5.0
4.0
3.0
2.0
1.0
f1 (ppm)

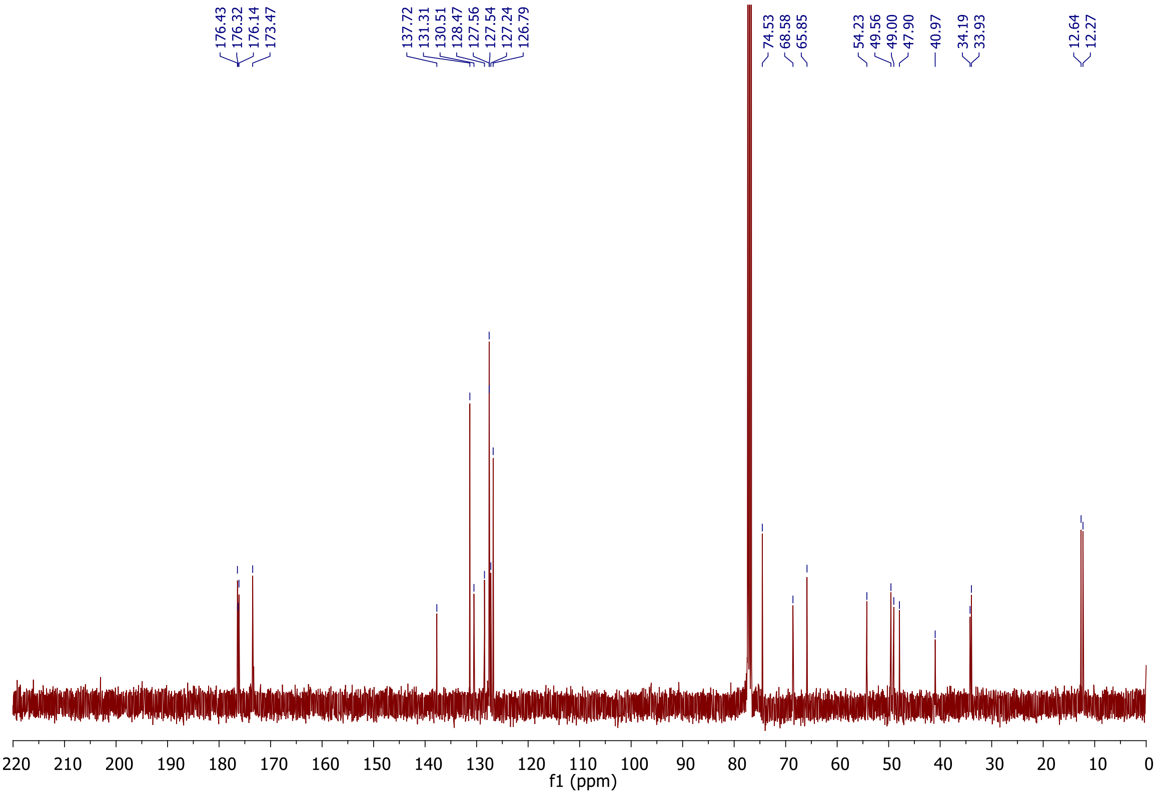
176.43
176.32
176.14
173.47
137.72
131.31
130.51
128.47
127.56
127.54
127.24
126.79
74.53
68.58
65.85
54.23
49.56
49.00
47.90
40.97
34.19
33.93
12.64
12.27
220
210
200
190
180
170
160
150
140
130
120
110
100
90
80
70
60
50
40
30
20
10
0
f1 (ppm)

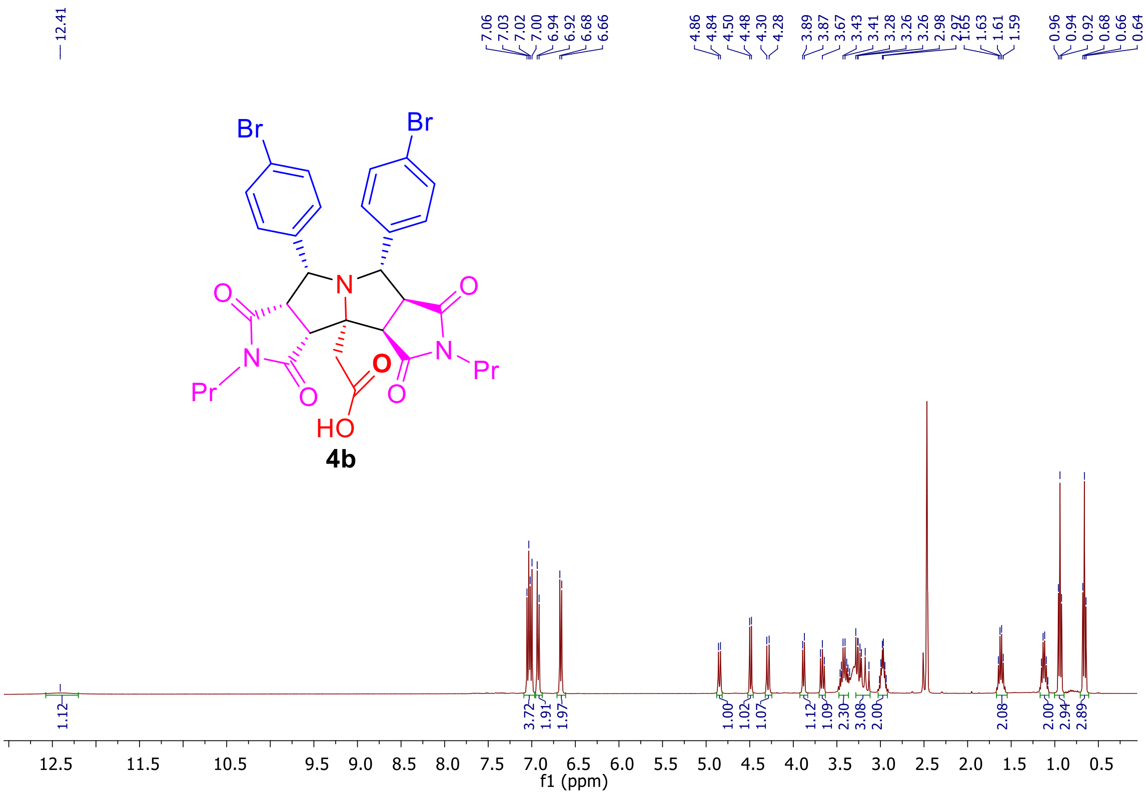

4b
12.41
7.06
7.03
7.02
7.00
6.94
6.92
6.68
6.66
4.86
4.84
4.50
4.48
4.30
4.28
3.89
3.87
3.67
3.43
3.41
3.28
3.26
3.26
2.98
2.97
1.65
1.63
1.61
1.59
0.96
0.94
0.92
0.68
0.66
0.64
1.12
3.72
1.91
1.97
1.00
1.02
1.07
1.12
1.09
2.30
3.08
2.00
2.08
2.00
2.94
2.89
f1 (ppm)

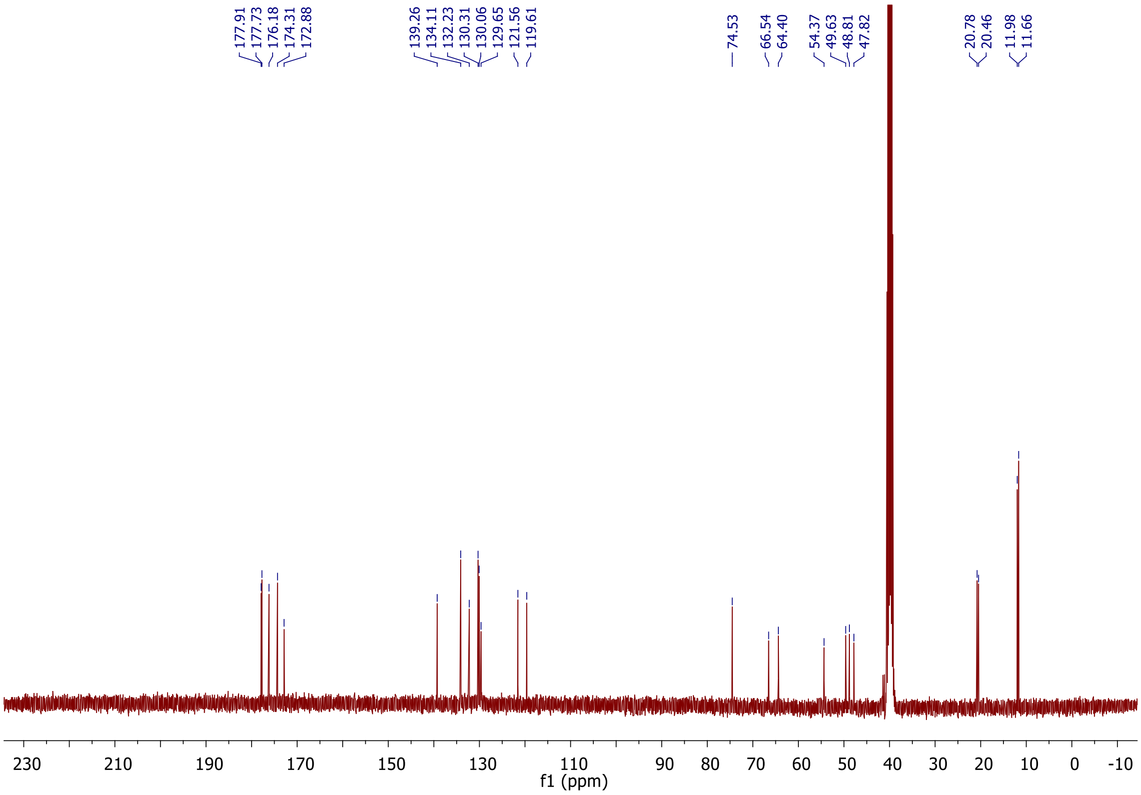

177.91
177.73
176.18
174.31
172.88
139.26
134.11
132.23
130.31
130.06
129.65
121.56
119.61
74.53
66.54
64.40
54.37
49.63
48.81
47.82
20.78
20.46
11.98
11.66
f1 (ppm)

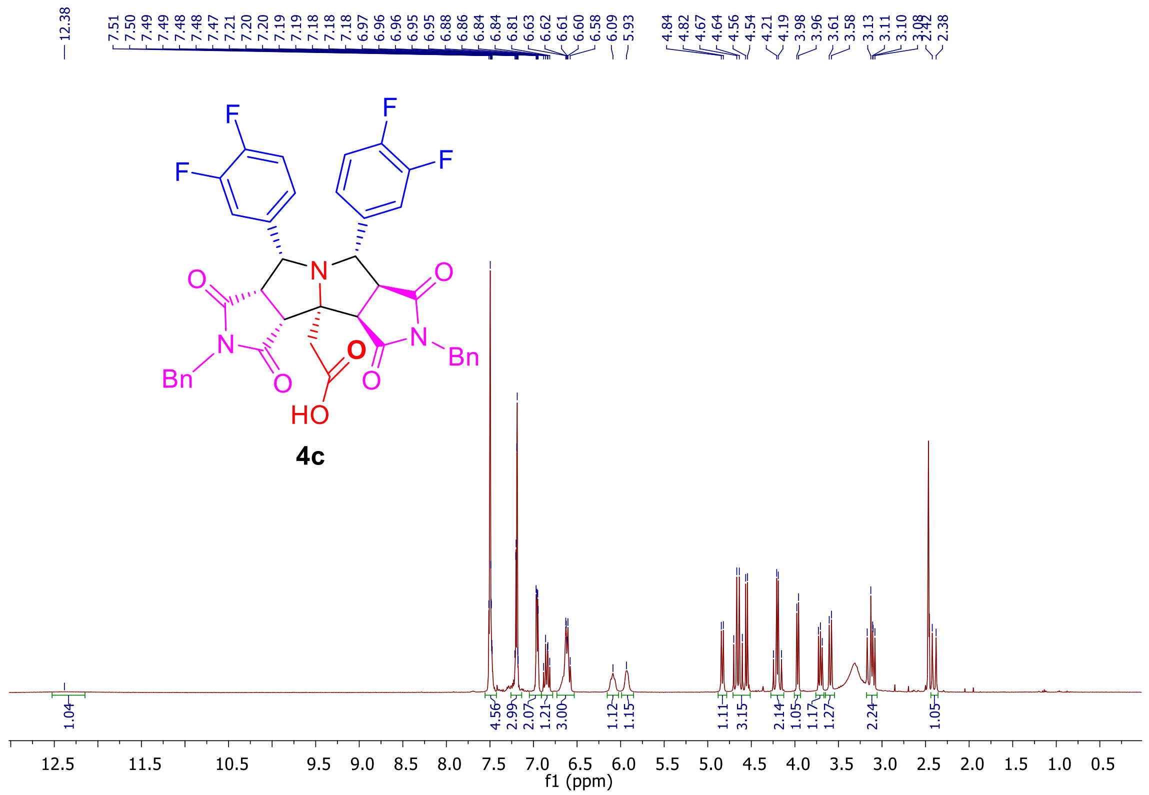
12.38
7.51 7.50 7.49 7.49 7.48 7.48 7.47 7.21 7.20 7.20 7.19 7.19 7.18 7.18 7.18 6.97 6.96 6.96 6.95 6.95 6.88 6.86 6.84 6.84 6.81 6.63 6.62 6.61 6.60 6.58 6.09 5.93
4.84 4.82 4.67 4.64 4.56 4.54 4.21 4.19 3.98 3.96 3.61 3.58 3.13 3.11 3.10 3.08 2.42 2.38
F
F
F
F
N
O
O
O
O
N
N
Bn
Bn
O
HO
4c
1.04 4.56 2.99 2.07 1.21 3.00 1.12 1.15 1.11 3.15 2.14 1.05 1.17 1.27 2.24 1.05
12.5 11.5 10.5 9.5 9.0 8.5 8.0 7.5 7.0 6.5 6.0 5.5 5.0 4.5 4.0 3.5 3.0 2.5 2.0 1.5 1.0 0.5
f1 (ppm)

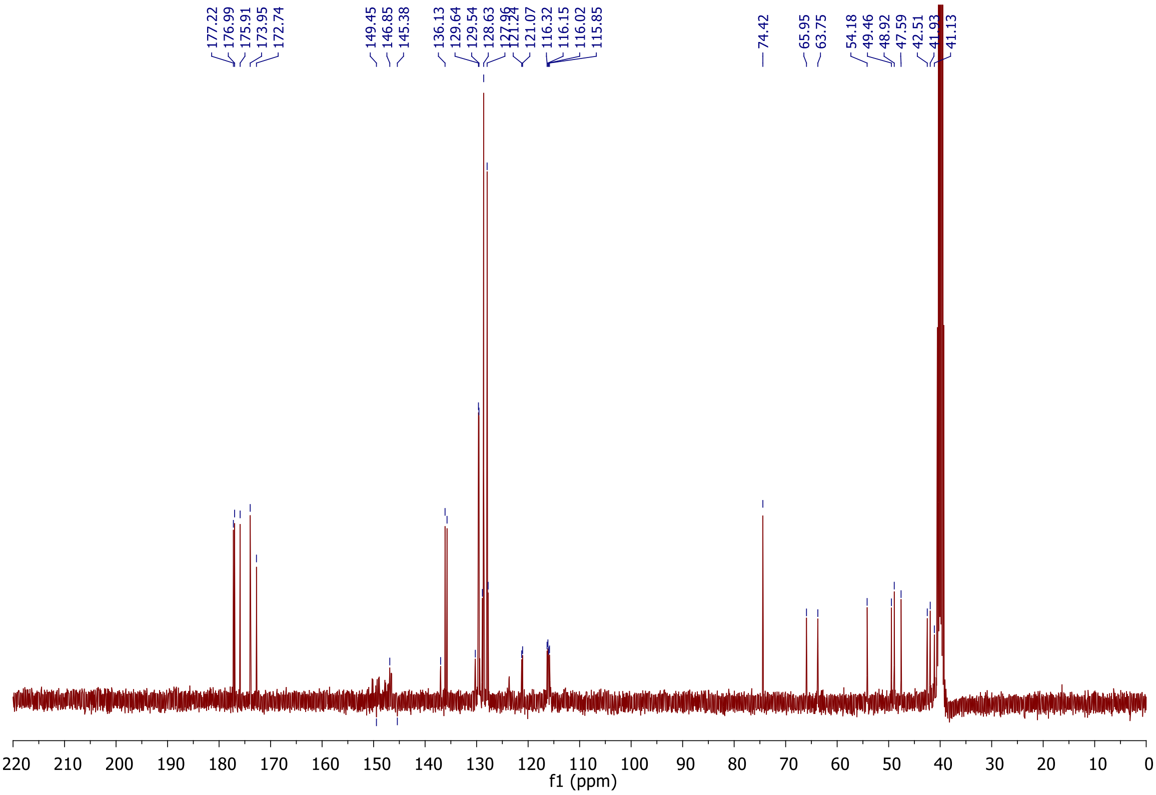
177.22 176.99 175.91 173.95 172.74
149.45 146.85 145.38
136.13 129.64 129.54 128.63 127.96 121.24 121.07 116.32 116.15 116.02 115.85
74.42
65.95 63.75
54.18 49.46 48.92 47.59 42.51 41.93 41.13
220 210 200 190 180 170 160 150 140 130 120 110 100 90 80 70 60 50 40 30 20 10 0
f1 (ppm)

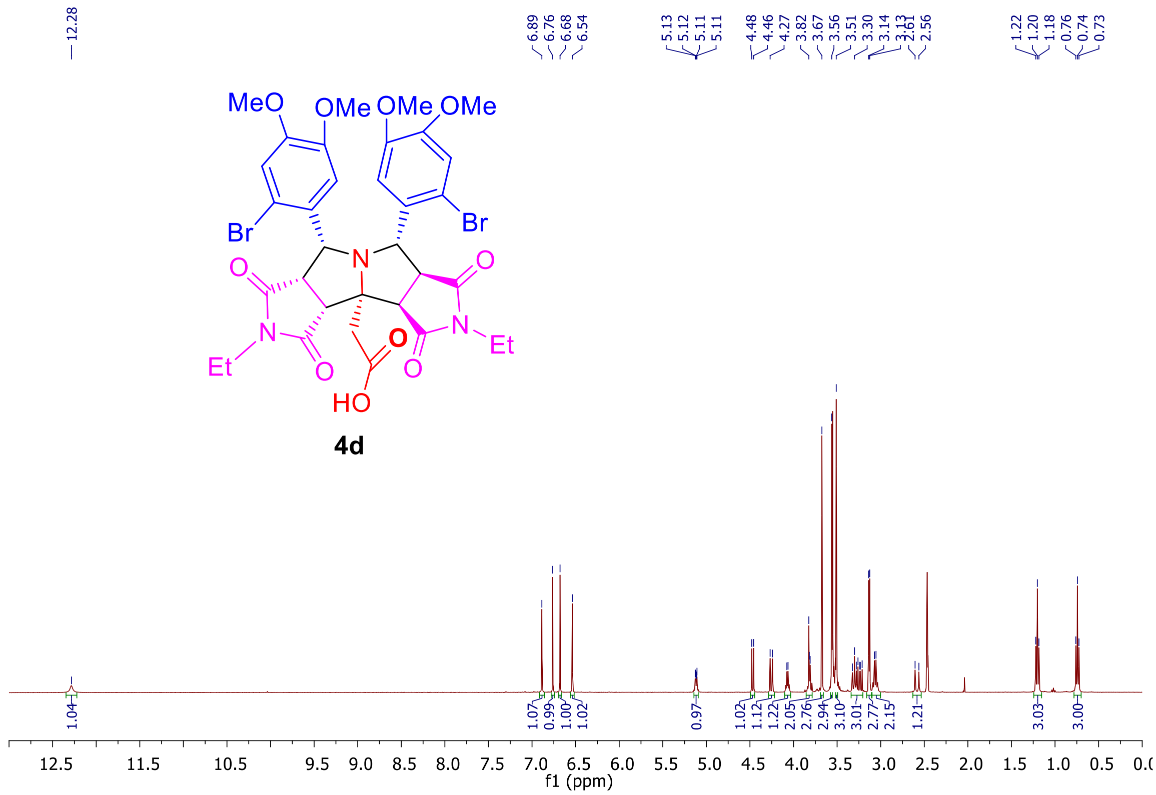
12.28
6.89
6.76
6.68
6.54
5.13
5.12
5.11
5.11
4.48
4.46
4.27
3.82
3.67
3.56
3.51
3.30
3.14
3.13
2.61
2.56
1.22
1.20
1.18
0.76
0.74
0.73
MeO
OMe
OMe
OMe
Br
Br
N
O
O
O
O
O
N
N
Et
Et
HO
1.04
1.07
0.99
1.00
1.02
0.97
1.02
1.12
1.22
2.05
2.76
2.94
3.10
3.01
2.77
2.15
1.21
3.03
3.00
12.5 11.5 10.5 9.5 9.0 8.5 8.0 7.5 7.0 6.5 6.0 5.5 5.0 4.5 4.0 3.5 3.0 2.5 2.0 1.5 1.0 0.5
f1 (ppm)

4d

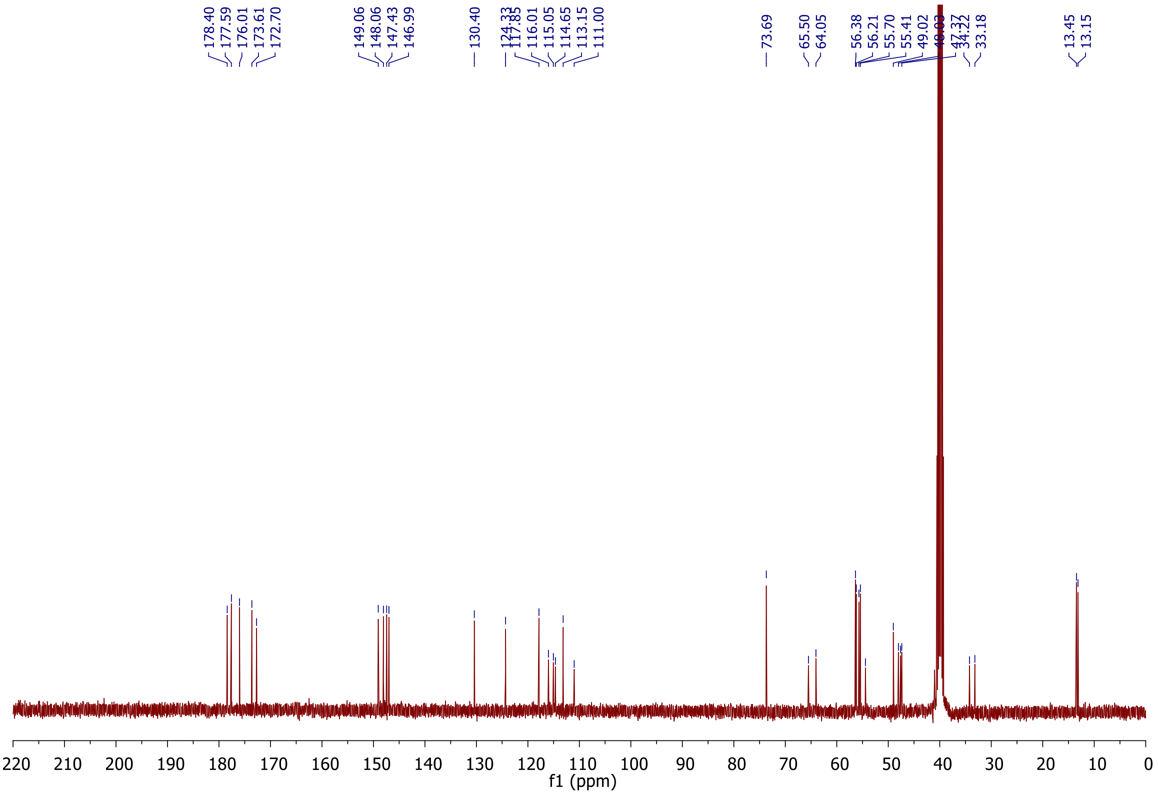
178.40
177.59
176.01
173.61
172.70
149.06
148.06
147.43
146.99
130.40
124.33
117.85
116.01
115.05
114.65
113.15
111.00
73.69
65.50
64.05
56.38
56.21
55.70
55.41
49.02
47.37
34.22
33.18
13.45
13.15
220 210 200 190 180 170 160 150 140 130 120 110 100 90 80 70 60 50 40 30 20 10 0
f1 (ppm)

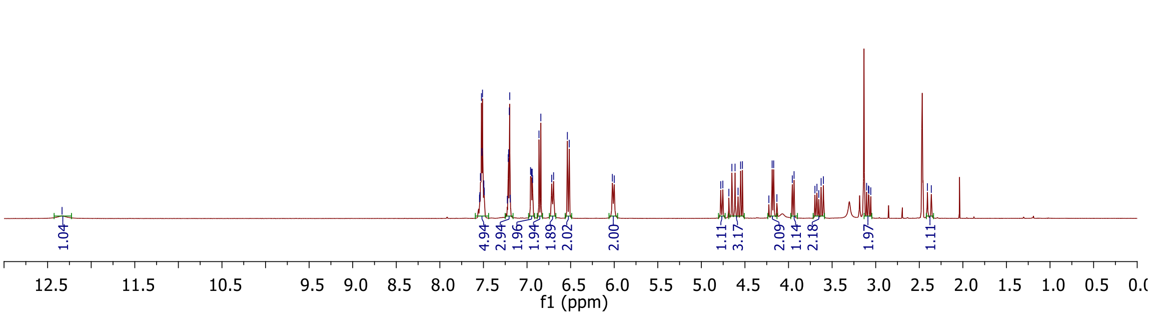

12.33
7.54
7.54
7.53
7.52
7.52
7.51
7.50
7.49
7.23
7.22
7.21
7.20
7.20
7.19
6.96
6.95
6.94
6.94
6.86
6.84
6.72
6.70
6.54
6.52
6.02
6.00
4.78
4.75
4.65
4.61
4.55
4.53
4.19
4.17
3.96
3.94
3.63
3.60
3.10
3.08
3.08
3.06
2.41
2.36
1.04
4.94
2.94
1.96
1.94
1.89
2.02
2.00
1.11
3.17
2.09
1.14
2.18
1.97
1.11
f1 (ppm)

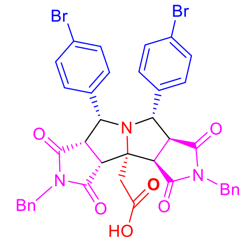

Br
Br
O
O
N
N
N
Bn
Bn
O
O
O
HO

4e

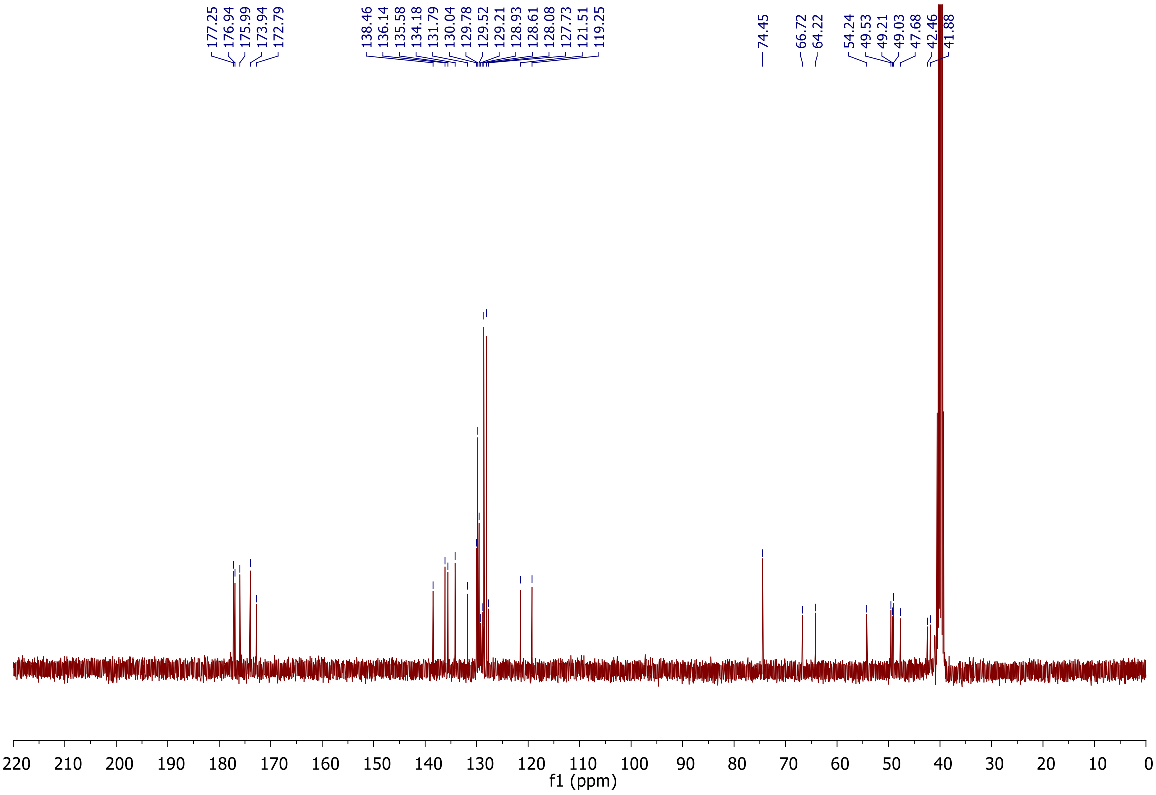

177.25
176.94
175.99
173.94
172.79
138.46
136.14
135.58
134.18
131.79
130.04
129.78
129.52
129.21
128.93
128.61
128.08
127.73
121.51
119.25
74.45
66.72
64.22
54.24
49.53
49.21
49.03
47.68
42.46
41.88
f1 (ppm)

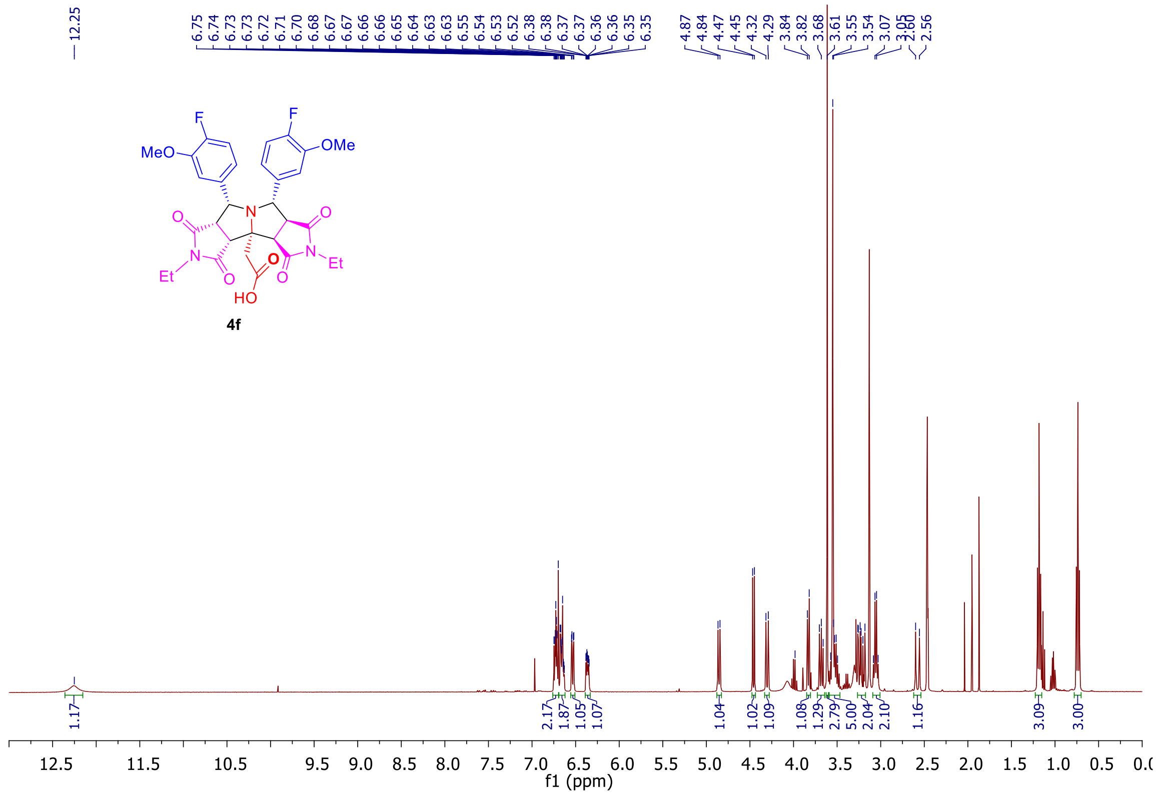
12.25
6.75 6.74 6.73 6.73 6.72 6.71 6.70 6.68 6.67 6.67 6.66 6.65 6.64 6.63 6.63 6.55 6.54 6.53 6.52 6.38 6.38 6.37 6.37 6.36 6.36 6.35 6.35
4.87 4.84 4.47 4.45 4.32 4.29 3.84 3.82 3.68 3.61 3.55 3.54 3.07 3.05 2.60 2.56
F
MeO
F
OMe
N
O
N
Et
O
O
N
Et
O
O
HO
4f
1.17
2.17 1.87 1.05 1.07
1.04 1.02 1.09 1.08 1.29 2.79 5.00 2.04 2.10 1.16 3.09 3.00
f1 (ppm)

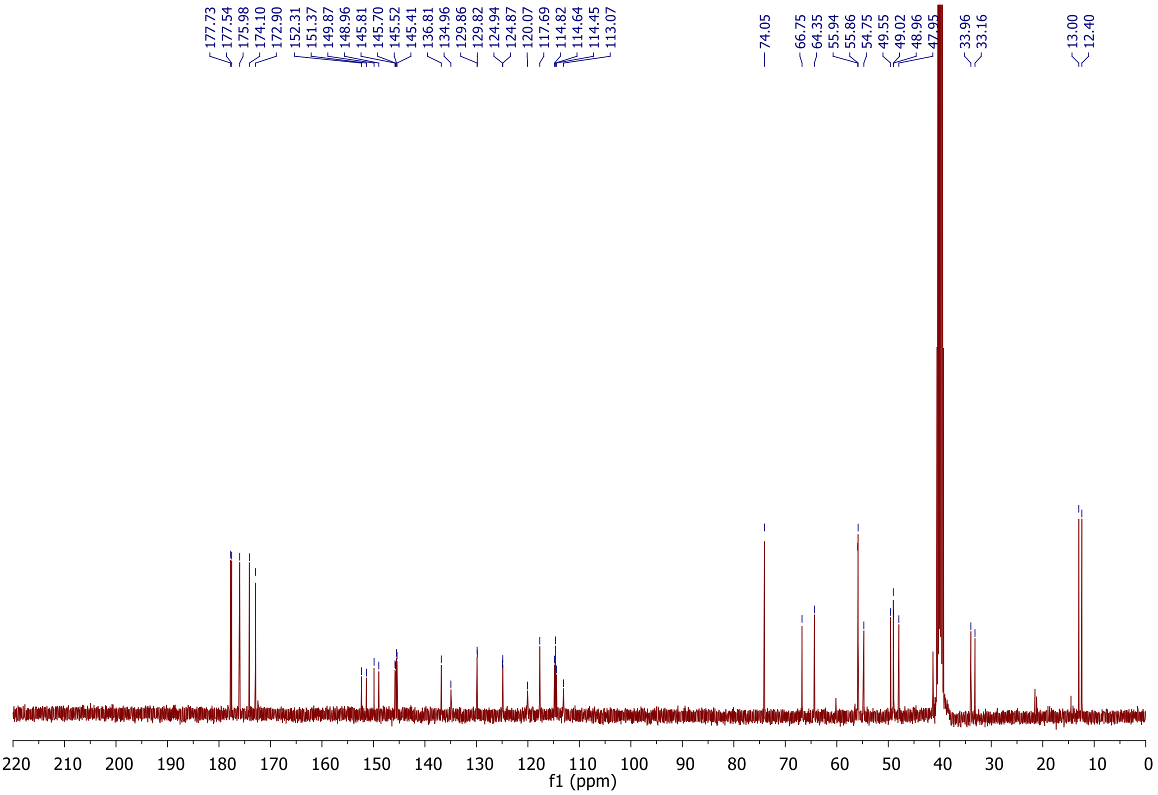
177.73 177.54 175.98 174.10 172.90 152.31 151.37 149.87 148.96 145.81 145.70 145.52 145.41 136.81 134.96 129.86 129.82 124.94 124.87 120.07 117.69 114.82 114.64 114.45 113.07
74.05
66.75 64.35 55.94 55.86 54.75 49.55 49.02 48.96 47.95
33.96 33.16
13.00 12.40
f1 (ppm)

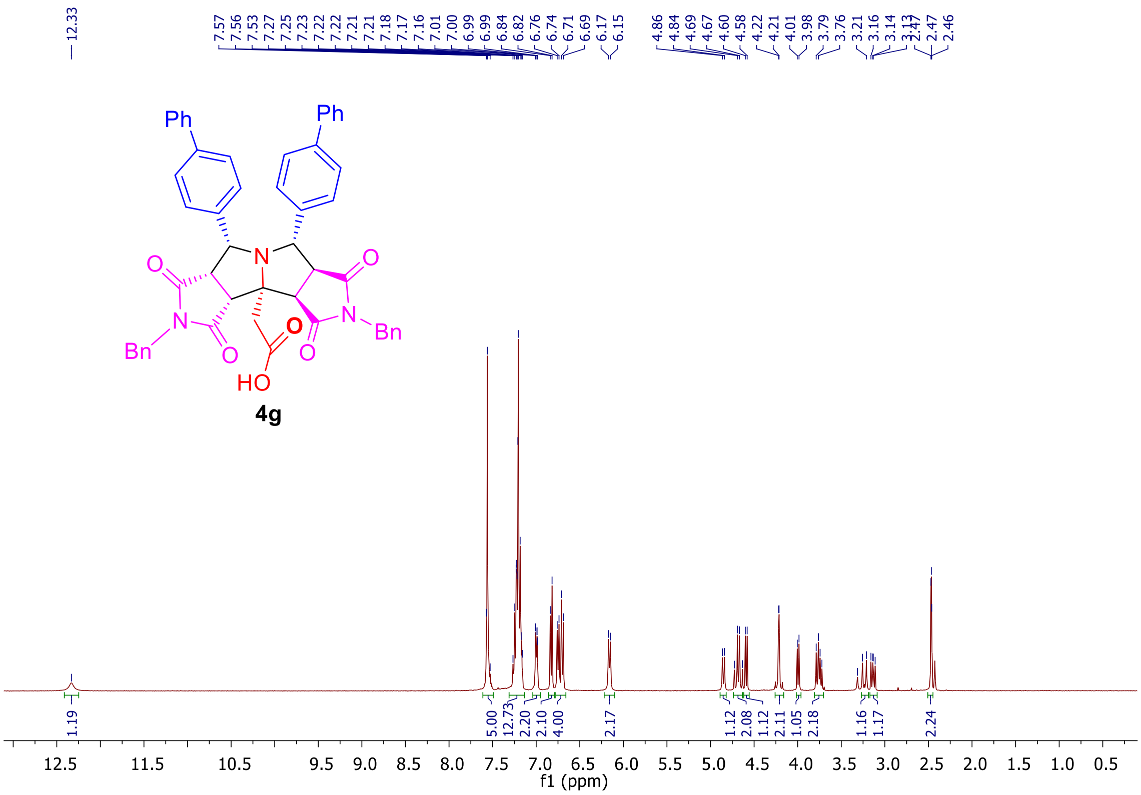

Ph
Ph
N
O
O
N
Bn
O
O
N
Bn
O
HO

4g

12.33
7.57
7.56
7.53
7.27
7.25
7.23
7.22
7.22
7.21
7.21
7.18
7.17
7.16
7.01
7.00
6.99
6.99
6.84
6.82
6.76
6.74
6.71
6.69
6.17
6.15
4.86
4.84
4.69
4.67
4.60
4.58
4.22
4.21
4.01
3.98
3.79
3.76
3.21
3.16
3.14
3.13
2.47
2.47
2.46
1.19
5.00
12.73
2.20
2.10
4.00
2.17
1.12
2.08
1.12
2.11
1.05
2.18
1.16
1.17
2.24
12.5
11.5
10.5
9.5
9.0
8.5
8.0
7.5
7.0
6.5
6.0
5.5
5.0
4.5
4.0
3.5
3.0
2.5
2.0
1.5
1.0
0.5
f1 (ppm)

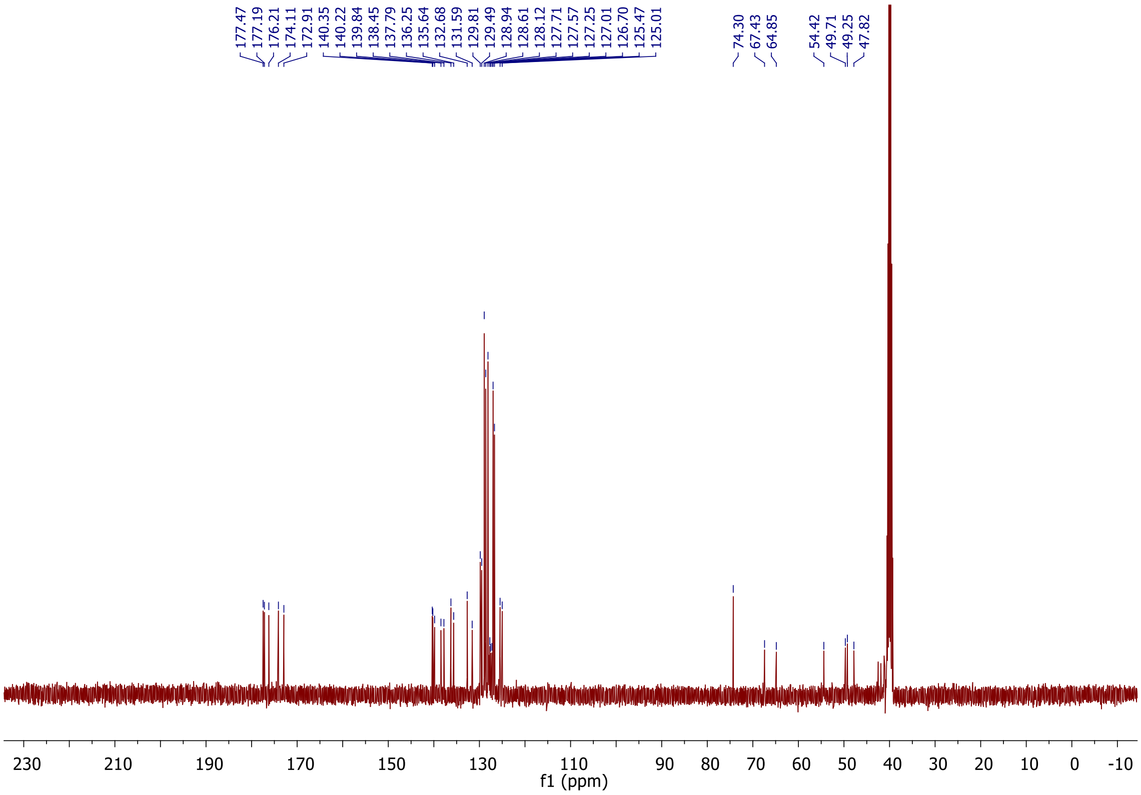

177.47
177.19
176.21
174.11
172.91
140.35
140.22
139.84
138.45
137.79
136.25
135.64
132.68
131.59
129.81
129.49
128.94
128.61
128.12
127.71
127.57
127.25
127.01
126.70
125.47
125.01
74.30
67.43
64.85
54.42
49.71
49.25
47.82
230
210
190
170
150
130
110
90
80
70
60
50
40
30
20
10
0
-10
f1 (ppm)

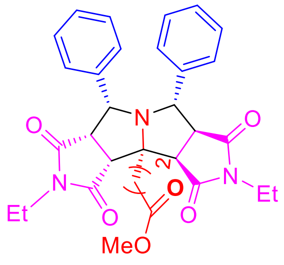
N
O
O
O
O
N
N
Et
Et
O
MeO
2

4j

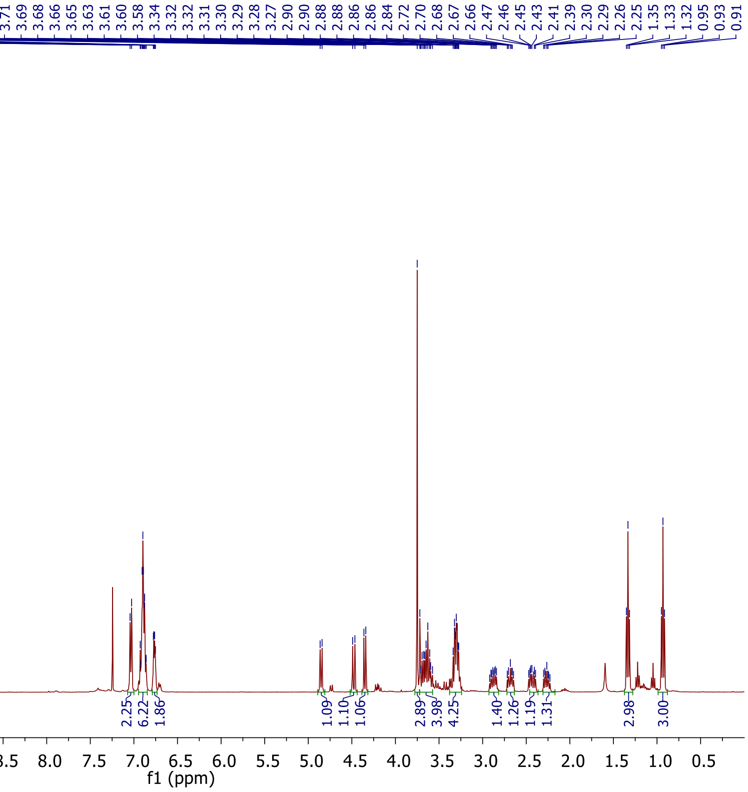
7.04 7.03 6.93 6.92 6.91 6.90 6.89 6.88 6.88 6.86 6.86 6.78 6.77 6.76 6.75 4.86 4.84 4.49 4.46 4.36 4.34 3.75 3.72 3.71 3.69 3.68 3.66 3.65 3.63 3.61 3.60 3.58 3.34 3.32 3.32 3.31 3.30 3.29 3.28 3.27 2.90 2.88 2.88 2.86 2.86 2.84 2.72 2.70 2.68 2.67 2.66 2.47 2.46 2.45 2.43 2.41 2.39 2.30 2.29 2.26 2.25 1.35 1.33 1.32 0.95 0.93 0.91
2.25 6.22 1.86 1.09 1.10 1.06 2.89 3.98 4.25 1.40 1.26 1.19 1.31 2.98 3.00
12.5 11.5 10.5 9.5 9.0 8.5 8.0 7.5 7.0 6.5 6.0 5.5 5.0 4.5 4.0 3.5 3.0 2.5 2.0 1.5 1.0 0.5
f1 (ppm)

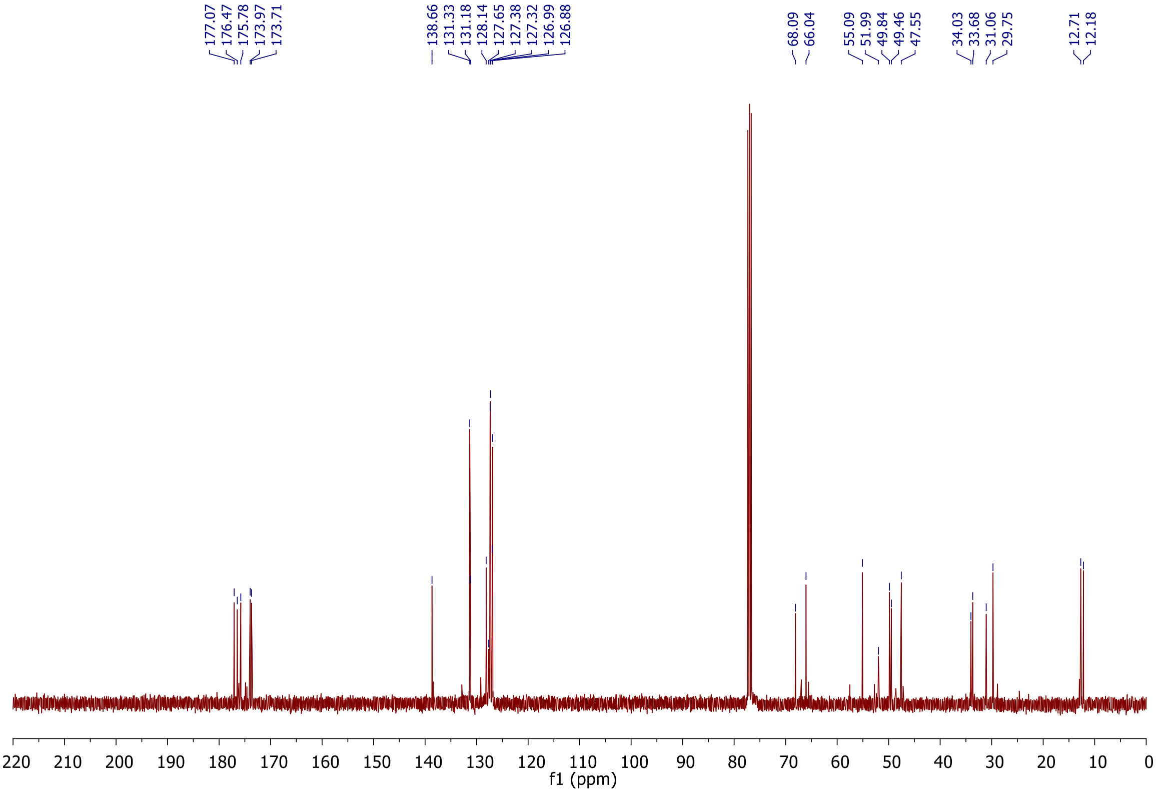
177.07 176.47 175.78 173.97 173.71 138.66 131.33 131.18 128.14 127.65 127.38 127.32 126.99 126.88 68.09 66.04 55.09 51.99 49.84 49.46 47.55 34.03 33.68 31.06 29.75 12.71 12.18
220 210 200 190 180 170 160 150 140 130 120 110 100 90 80 70 60 50 40 30 20 10 0
f1 (ppm)

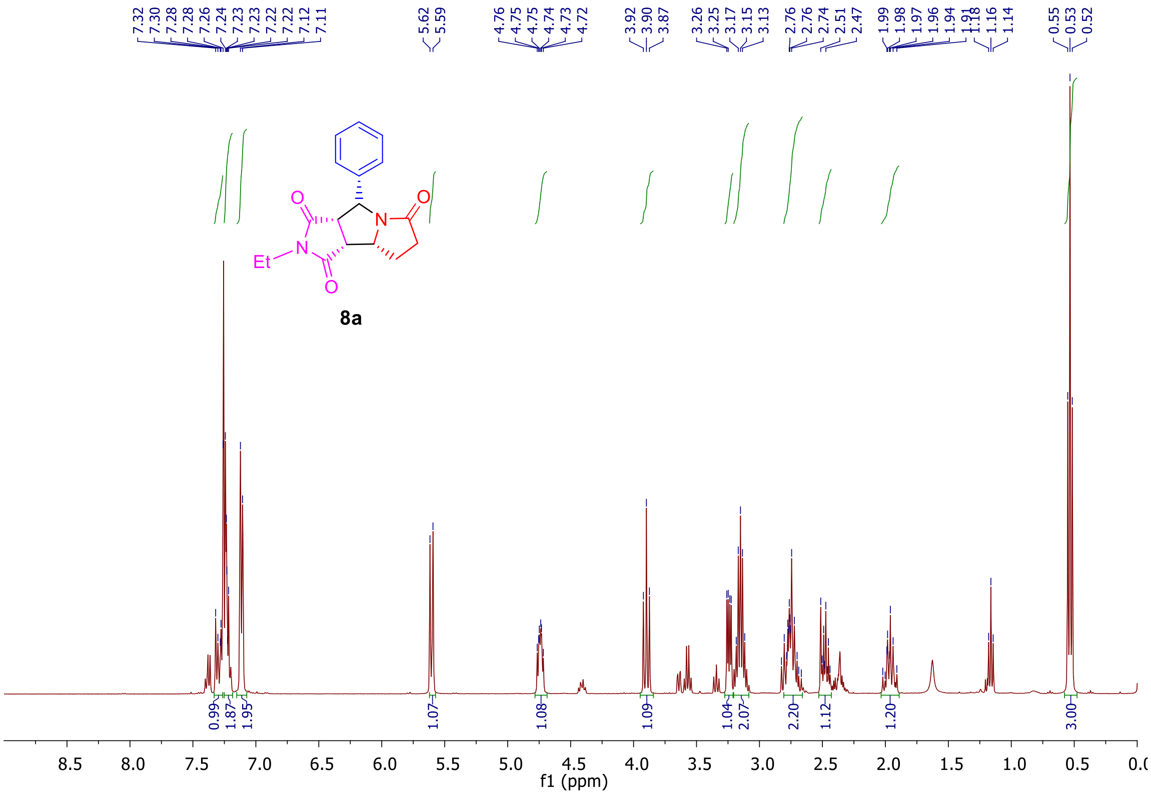
7.32 7.30 7.28 7.28 7.26 7.24 7.23 7.23 7.22 7.22 7.12 7.11
5.62 5.59
4.76 4.75 4.75 4.74 4.73 4.72
3.92 3.90 3.87
3.26 3.25 3.17 3.15 3.13
2.76 2.76 2.74 2.51 2.47
1.99 1.98 1.97 1.96 1.94 1.91 1.18 1.16 1.14
0.55 0.53 0.52
O
N
O
Et
N
O
8a
0.99 1.87 1.95 1.07 1.08 1.09 1.04 2.07 2.20 1.12 1.20 3.00
8.5 8.0 7.5 7.0 6.5 6.0 5.5 5.0 4.5 4.0 3.5 3.0 2.5 2.0 1.5 1.0 0.5
f1 (ppm)

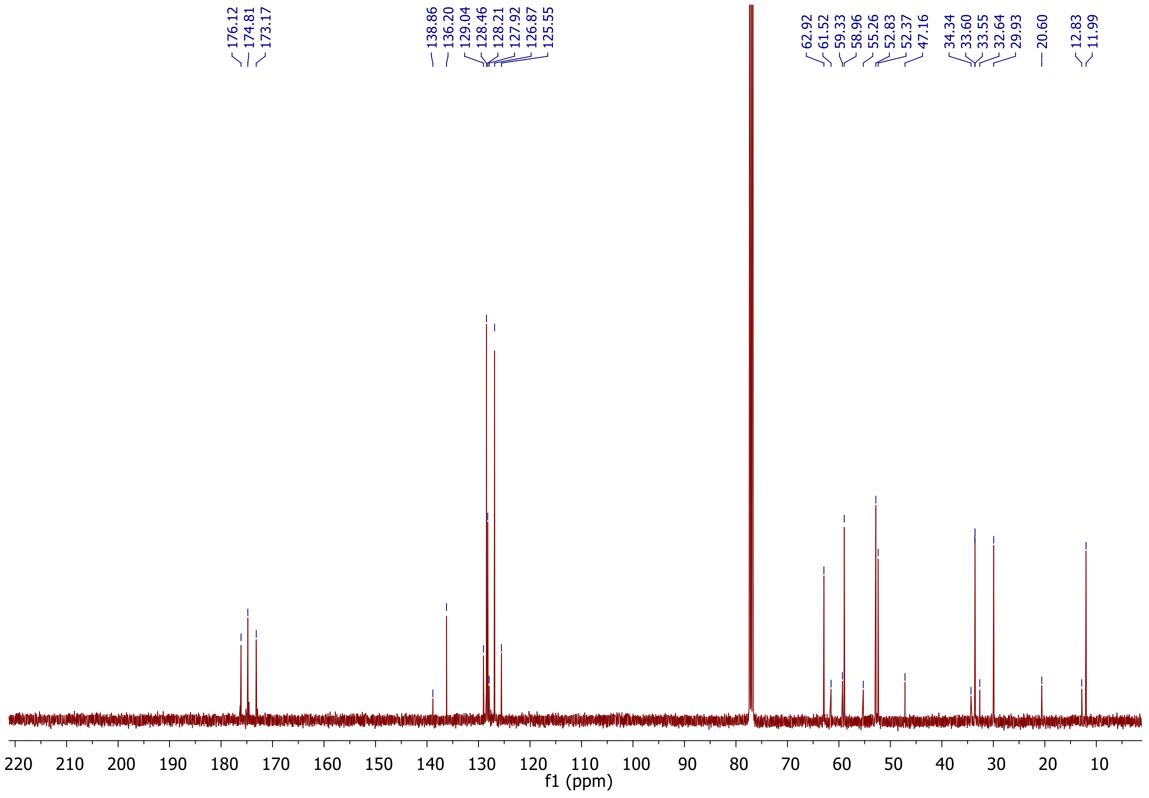
176.12 174.81 173.17
138.86 136.20 129.04 128.46 128.21 127.92 126.87 125.55
62.92 61.52 59.33 58.96 55.26 52.83 52.37 47.16
34.34 33.60 33.55 32.64 29.93
20.60
12.83 11.99
220 210 200 190 180 170 160 150 140 130 120 110 100 90 80 70 60 50 40 30 20 10
f1 (ppm)

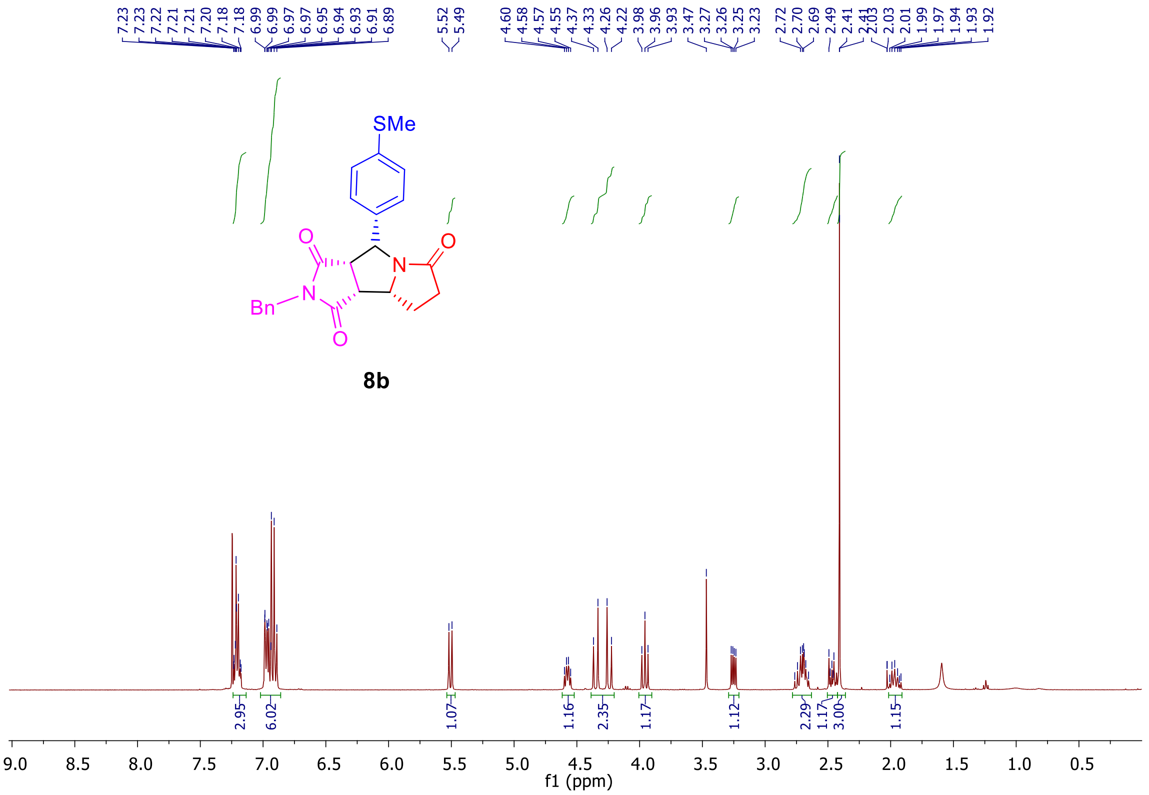
7.23 7.23 7.22 7.21 7.21 7.20 7.18 7.18 6.99 6.99 6.97 6.97 6.95 6.94 6.93 6.91 6.89
5.52 5.49
4.60 4.58 4.57 4.55 4.37 4.33 4.26 4.22 3.98 3.96 3.93 3.47 3.27 3.26 3.25 3.23
2.72 2.70 2.69 2.49 2.41 2.41 2.03 2.03 2.01 1.99 1.97 1.94 1.93 1.92
SMe
O
N
Bn
O
8b
2.95 6.02 1.07 1.16 2.35 1.17 1.12 2.29 1.17 3.00 1.15
9.0 8.5 8.0 7.5 7.0 6.5 6.0 5.5 5.0 4.5 4.0 3.5 3.0 2.5 2.0 1.5 1.0 0.5
f1 (ppm)

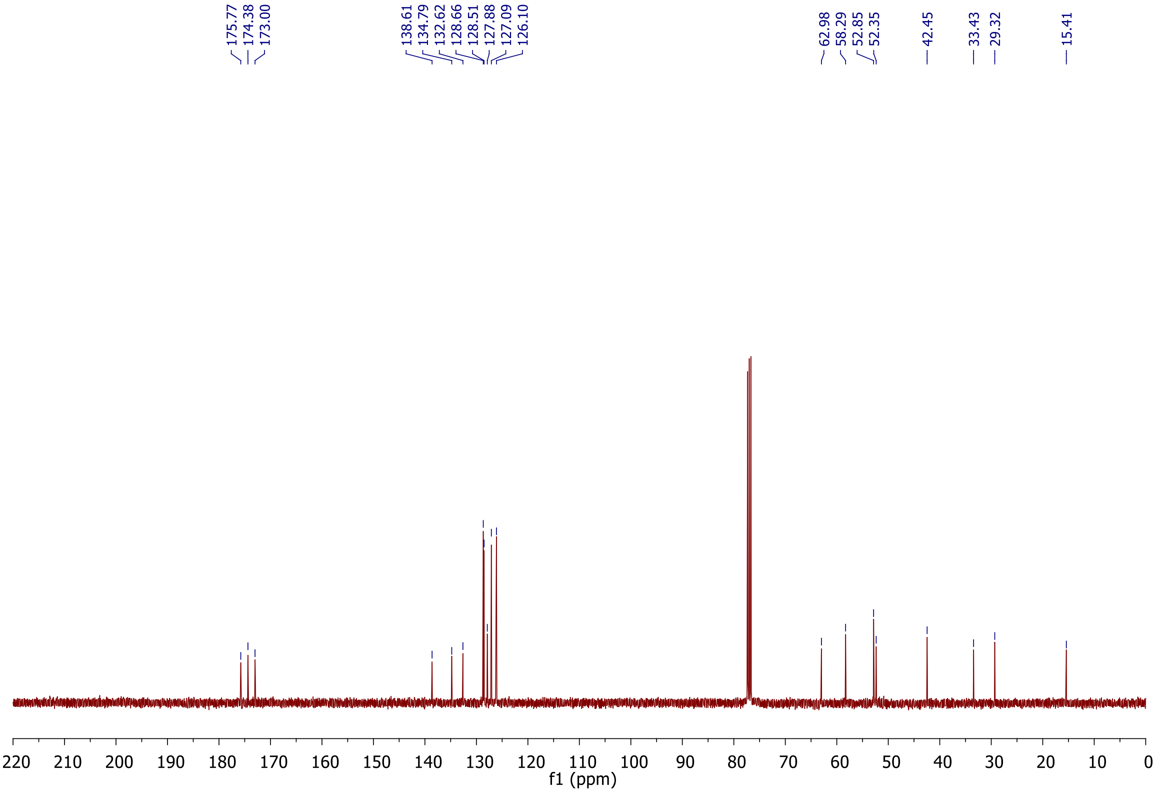
175.77 174.38 173.00
138.61 134.79 132.62 128.66 128.51 127.88 127.09 126.10
62.98 58.29 52.85 52.35
42.45
33.43 29.32
15.41
220 210 200 190 180 170 160 150 140 130 120 110 100 90 80 70 60 50 40 30 20 10 0
f1 (ppm)

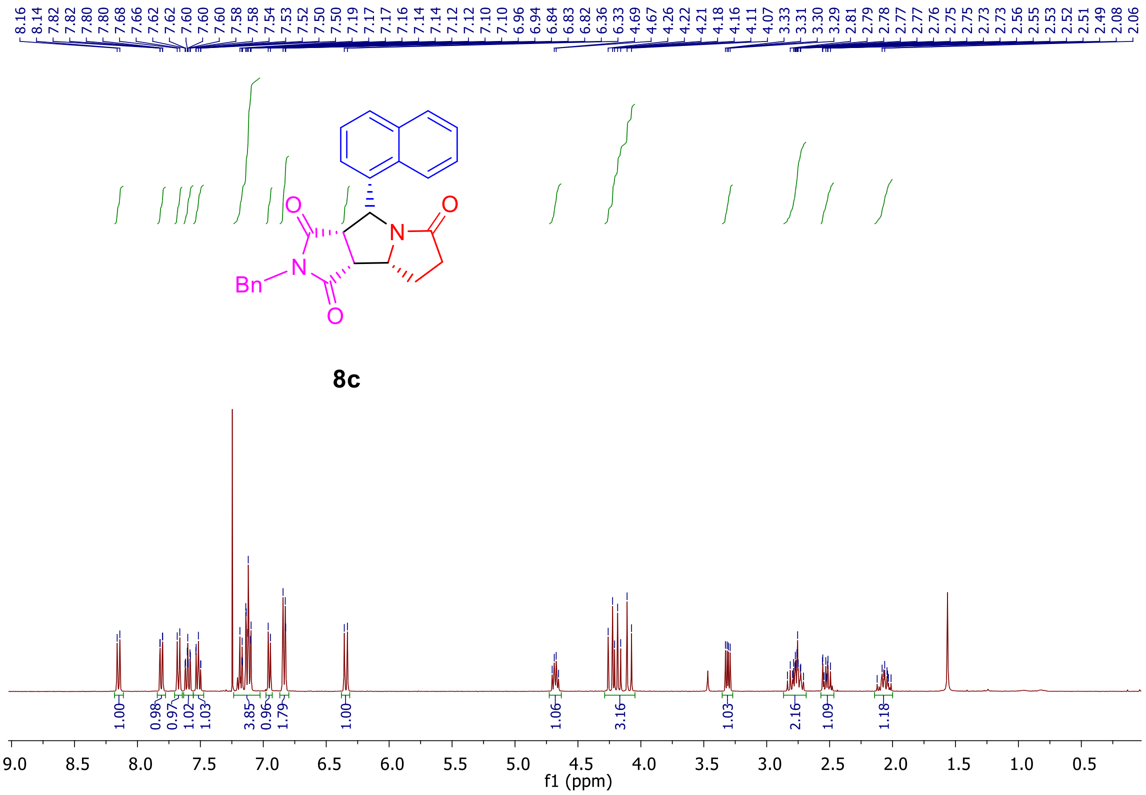
8.16
8.14
7.82
7.82
7.80
7.80
7.68
7.66
7.62
7.60
7.60
7.60
7.58
7.58
7.54
7.53
7.52
7.50
7.19
7.17
7.17
7.16
7.14
7.14
7.12
7.12
7.10
7.10
6.96
6.94
6.84
6.83
6.82
6.36
6.33
4.69
4.67
4.26
4.22
4.21
4.18
4.16
4.11
4.07
3.33
3.31
3.30
3.29
2.81
2.79
2.78
2.77
2.77
2.76
2.75
2.75
2.73
2.73
2.56
2.55
2.53
2.52
2.51
2.49
2.08
2.06
O
N
O
Bn
N
O
O
8c
1.00
0.98
0.97
1.02
1.03
3.85
0.96
1.79
1.00
1.06
3.16
1.03
2.16
1.09
1.18
9.0
8.5
8.0
7.5
7.0
6.5
6.0
5.5
5.0
4.5
4.0
3.5
3.0
2.5
2.0
1.5
1.0
0.5
f1 (ppm)

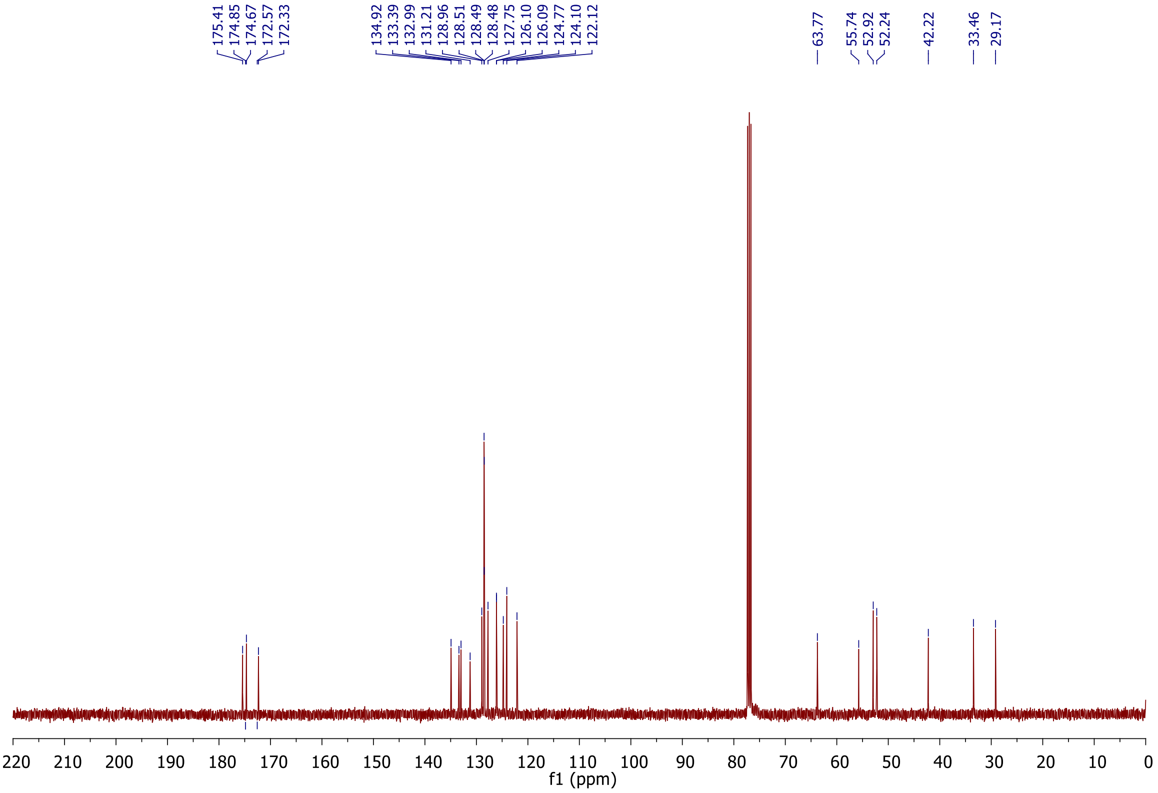
175.41
174.85
174.67
172.57
172.33
134.92
133.39
132.99
131.21
128.96
128.51
128.49
128.48
127.75
126.10
126.09
124.77
124.10
122.12
63.77
55.74
52.92
52.24
42.22
33.46
29.17
220
210
200
190
180
170
160
150
140
130
120
110
100
90
80
70
60
50
40
30
20
10
0
f1 (ppm)

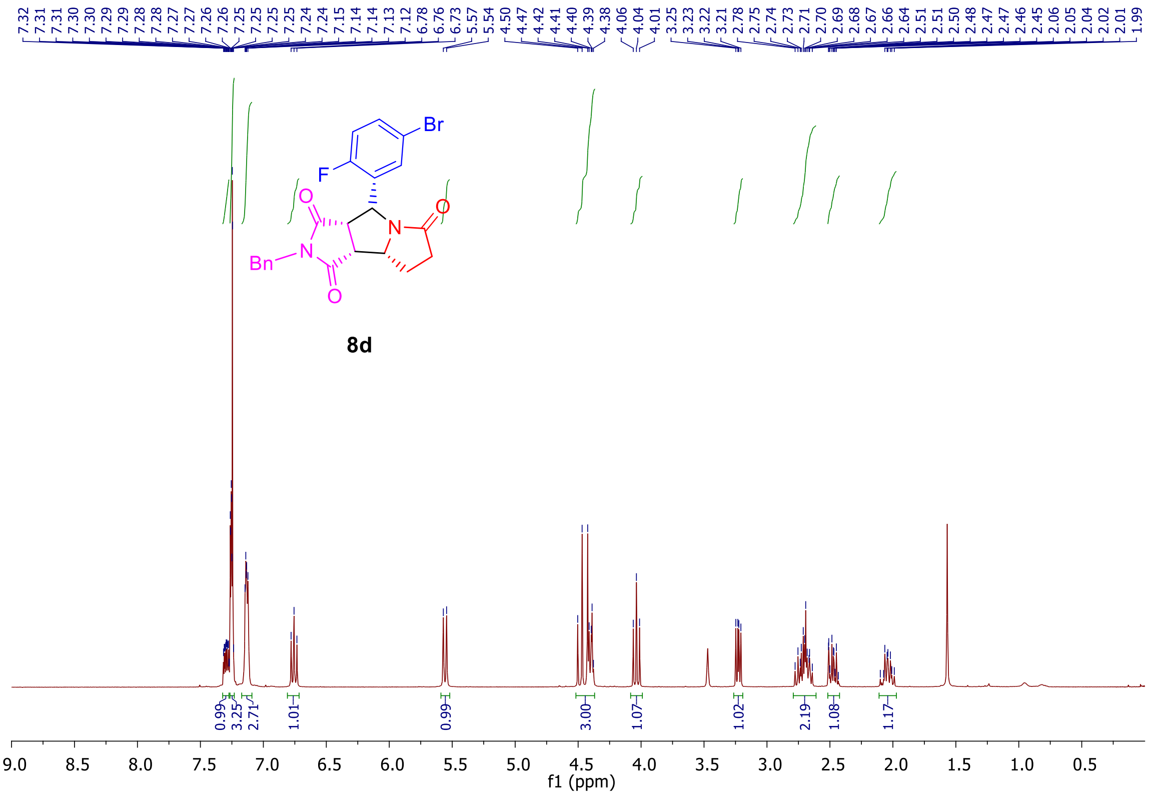

8d

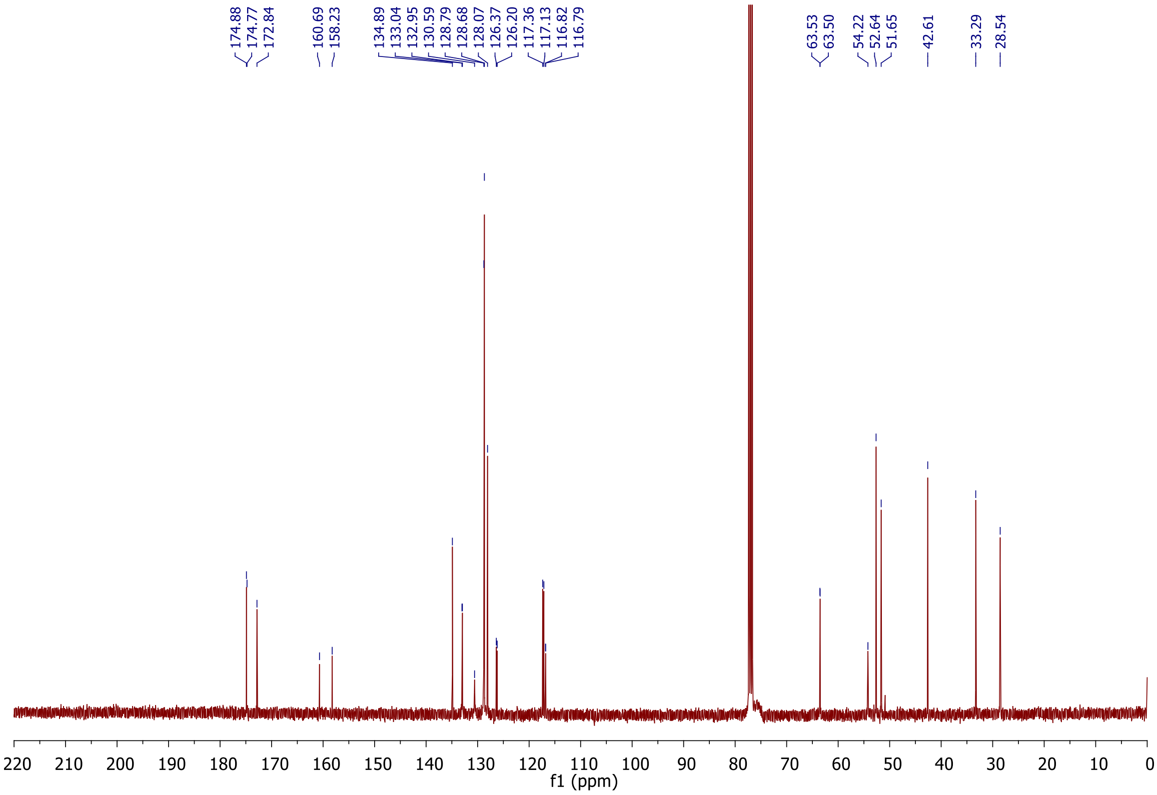

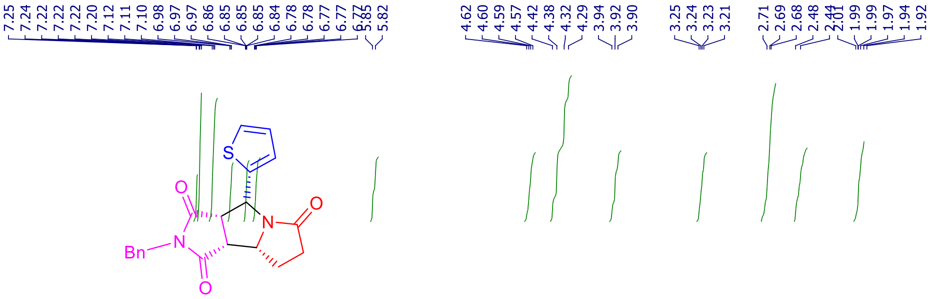

8e

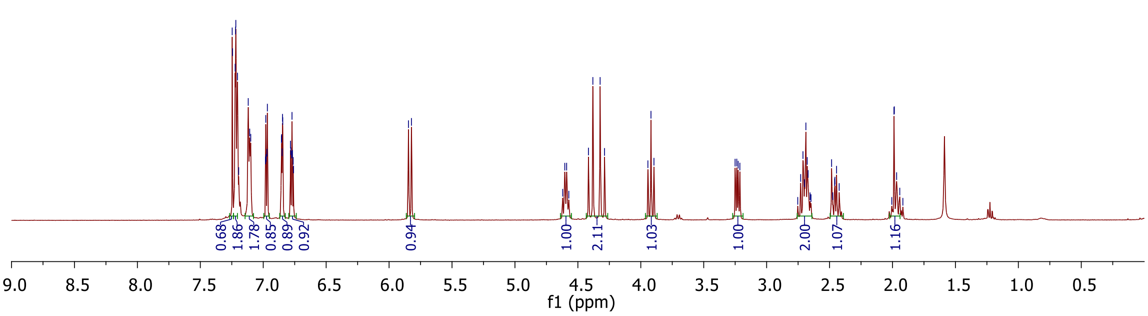
7.25
7.24
7.22
7.22
7.22
7.20
7.12
7.11
7.10
6.98
6.97
6.97
6.86
6.85
6.85
6.85
6.84
6.78
6.78
6.77
6.77
5.85
5.82
4.62
4.60
4.59
4.57
4.42
4.38
4.32
4.29
3.94
3.92
3.90
3.25
3.24
3.23
3.21
2.71
2.69
2.68
2.48
2.44
2.01
1.99
1.99
1.97
1.94
1.92
0.68
1.86
1.78
0.85
0.89
0.92
0.94
1.00
2.11
1.03
1.00
2.00
1.07
1.16
9.0 8.5 8.0 7.5 7.0 6.5 6.0 5.5 5.0 4.5 4.0 3.5 3.0 2.5 2.0 1.5 1.0 0.5
f1 (ppm)

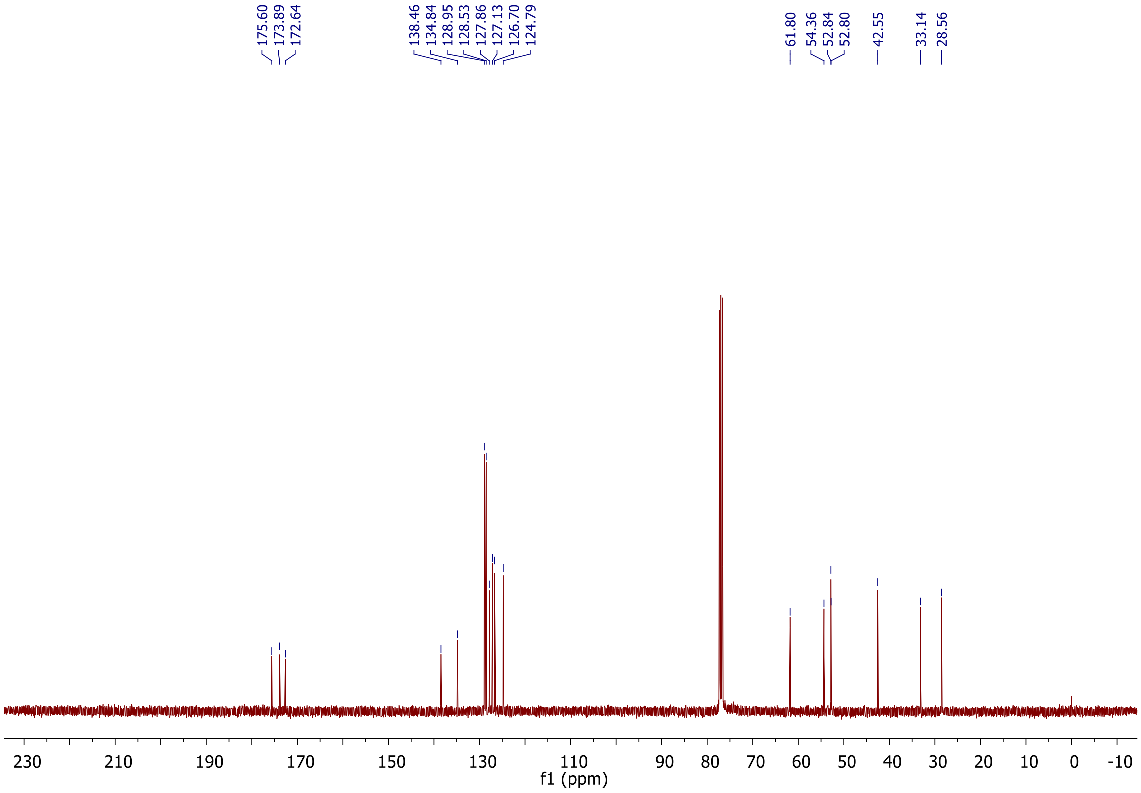
175.60
173.89
172.64
138.46
134.84
128.95
128.53
127.86
127.13
126.70
124.79
61.80
54.36
52.84
52.80
42.55
33.14
28.56
230 210 190 170 150 130 110 90 80 70 60 50 40 30 20 10 0 -10
f1 (ppm)
